# Process and Space

**DOI:** 10.3390/e28060683

**Published:** 2026-06-13

**Authors:** William Sulis

**Affiliations:** Collective Intelligence Laboratory, McMaster University, Hamilton, ON N0A 1E0, Canada; sulisw@mcmaster.ca; Tel.: +1-905-772-7218

**Keywords:** process algebra, process, generativity, temporal distinction, spatial distinction, spacetime history, mixed multigraphs, transience, local becoming, contextuality, locality, realism

## Abstract

From the perspective of process, time may be viewed as that which marks the occurrence of change, as previously proposed by this author. In contrast, spatial distinctions may be viewed as enabling the individuation and counting of events generated by processes. Following a conceptual discussion of Whitehead’s process theory, temporal distinctions, and spatial distinctions, a formal model of spacetime as history is presented based upon process *actions* as generators of spacetime, and a new geometric concept of ‘*thereness*’ is introduced. Each process action propagates information to the next generation (time) and to a particular ‘there’ (space). This generates a mixed multigraph where the directed subgraph represents the timelike component (causal propagation of information) and the undirected subgraph represents the spacelike component (informational correlations arising from common causes). A spatial *position* is an equivalence class of generated events; thus, it is emergent. Each spacetime is local to its generating process, consistent with the concept of local becoming proposed by Arthur. If the set of process actions forms a commutative monoid, then the resulting spacetime takes the form of a discrete lattice. It is speculated that the intransitivity and incompleteness of the spacelike subgraph may be linked to the presence of contextuality.

## 1. Introduction

The present work is part of a long-term project to develop a locally causal, contextual, generative, realist framework within which to situate a unified theory for complex dynamical systems generally—not just physics in particular. Within this framework, quantum mechanics, classical mechanics, and general relativity are viewed as effective theories at their respective scales and not general theories holding across all scales. It is anti-reductionist in the sense that it does not give primacy to one scale from which all others can be derived. It embraces Anderson’s dictum ‘more is different’; takes openness, self-organization, contextuality, and emergence to be ubiquitous; and views the changes in dynamics observed across scales as manifestations of emergence. Here, contextuality may take one of two forms: Type I contextuality, referring to having context-dependent probabilities, or Type II contextuality, referring to having correlations that violate some inequality, such as Bell, CHSH, Leggett–Garg, or Dzhafarov, among others. This framework is proposed as a general language within which to describe complex systems at any scale, akin to the role of category theory in mathematics and physics [1]. It does not constitute a specific model of complex systems. The framework is grounded philosophically upon an interpretation (probably quite idiosyncratic) of Whitehead’s theory of process [2] and mathematically on a formal system called the process algebra [3], inspired by combinatorial game theory, interpolation theory, and multigraphs.

The matter of time was presented in [4,5]. Since time and space are inextricably intertwined, no discussion of fundamental relations can move forward without consideration of space—hence, the focus of this paper. In contrast to standard approaches to time and space, the process approach assumes that space and time are emergent entities that are generated by process. Before this idea can be exploited, it is first necessary to understand its implications for the mathematical structures that represent such generation. The Whiteheadean concept of process encompasses much more than the usual physical understanding, and the resulting concept of space is more general than that of a traditional physical spacetime, more akin to a mathematician’s use of the term ‘space’. To encompass the greater generality of process, the concept of spatial distinction is introduced, which expresses this generalization. An axiomatization of process-generated spacetime based on a primitive notion of ‘thereness’ is then developed, and some of its implications are explored. In this paper, the term ‘spacetime’ refers to any formal structure in which some components convey information about temporal distinctions, while others convey information about spatial distinctions. The notion of position will be seen to be an emergent construct. An important question is how the algebraic structure of process actions relates to the geometric structure of the generated spacetime.

The concept of spacetime has evolved over time. Referring to physical spacetime, Newton described an absolute spacetime, later identified with real, four-dimensional, Euclidean manifolds, in which events occur. Space and time were independent entities, viewed as containers for events. With the development of special relativity, space and time became relative to the inertial motion of observers, no longer independent but separable as dimensions, and spacetime became represented as real, four-dimensional, Minkowski manifolds. Nevertheless, space and time remained containers for events. The study of non-inertial motion led to the equivalence principle and later to general relativity. Space and time remained relative but now became entangled in a unified spacetime. This spacetime became an entity in its own right, shaping and being shaped by the events that occurred within it. It presents a picture of a universe as a frozen block, in which everything past, present, and future co-exists. However, it may also be viewed as the *history* of a universe, rather than as depicting the universe itself.

Process theory, as conceived by Whitehead, provides a radical alternative view to the above. Based on the concept of organism, it views primitive events, called *actual occasions*, as coming into being, persisting transiently, and then fading away. *Spacetime becomes epistemological—a history, not an ontology*. Shimony [6] considered Whitehead’s process theory in the context of fundamental physics. Initially positive towards it, he later rejected it, in part because of a misunderstanding that Whitehead’s actual occasions meant that electrons possess parts, whereas they mean that electrons are generated moment to moment. Processes, nevertheless, share many characteristics with quantum fields, as noted by Epperson [7]. Process ideas were also explored by notable thinkers such as Bohm, Hartshorne, and Prigogine [8]. Whitehead [2] and Malin [9] emphasized that processes create their actual occasions atemporally and thus *generate* spacetime, as opposed to arising *in* spacetime. Tanaka [10] also affirmed this view. In addition, Tanaka demonstrated that indivisibility, such as is exhibited by Whitehead’s actual occasions, leads directly to the possibility of violations of Bell-type inequalities (conversely, divisibility implies that Bell-type inequalities are satisfied). Chew [11] suggested that actual occasions, which he termed ‘pre-events’, would manifest over an interval Planck time in length.

Finkelstein [12] and Hiley [13] have both explored the use of algebraic tools to apply process ideas to physics—in particular, the use of Clifford algebras. The process algebra is not a Clifford algebra, but the insight that processes might be best studied via algebra rather than by geometry or analysis is notable. For all of its radicalism, Riffert [14] argued that Whitehead’s process theory is a legitimate scientific metaphysics as defined by Bunge. Moreover, he pointed out that Karl Popper, who initially rejected Whitehead’s philosophy as being ‘irrationalist’, later wrote, ‘Today the universe does not appear as an assemblage of things, but as a multitude of interactions and processes’ (as, in particular, Whitehead had stressed) [14]. Nobo [15] undertook a detailed philosophical analysis of Whitehead’s concept of actual occasion, emphasizing its discrete, holistic, extended, and informational nature.

Process theory and models are not the only possible approaches to an emergent spacetime. Three candidate approaches to quantum gravity, namely causal sets, loop quantum gravity, and string theory, suggest that spacetime can emerge even though there may be no spatiotemporal relations at the lowest level. Thus, the idea of an atemporal generation of actual occasions by process seems not so outlandish after all. Huggett and Wüthrich [16] argued that these three theories are best understood as representing a block universe [16,17]. The Process Algebra approach proposes that what is generated is a local compound present (no simultaneity is assumed), and spacetime provides merely a history of process actions. It is interesting that, prior to his elaboration of his theory of process, Whitehead had developed his own version of general relativity that included a form of simultaneity and a flat spacetime structure [18]. Ultimately, however, it failed later experimental tests of general relativity, and it appears that he never revisited his earlier model.

The nature of a generated time within a process worldview was presented in [4,5]. This paper extends this work by exploring the concept of a generated spacetime, focusing upon the nature of space from a process perspective. The goal here is not to recreate what we think we already know—that is, the usual structure of relativistic spacetimes—which is not possible anyway since the products of process are discrete and finite. Instead, the goal is to explore as fully as possible the implications of the process view and then compare these against empirical evidence. Thus, in this paper, I take a generic process and examine the general form that emergent spatiotemporal structures take as generated by process.

This paper is divided into three parts. Part 1, consisting of Section 2, Section 3, Section 4 and Section 5, provides a brief summary of Whitehead’s process theory and of the path integral model as an example of the process algebra applied to non-relativistic quantum mechanics (NRQM), as well as a conceptual discussion of the idea of generated time and space within a process framework, with a particular emphasis on the concepts of temporal and spatial distinctions. Readers who are familiar with process ideas (or averse to philosophically inclined discourse) can skip these sections and proceed to Part 2, consisting of Section 6, Section 7, Section 8, Section 9 and Section 10, which discuss the implications of such a generated spacetime from a mathematical perspective. In the Appendix A, the reader will find some basic definitions of the process algebra, together with a brief description of a generic process model. More details can be found in [3].

## 2. Whitehead and Process

Why should anyone be interested in approaching complex systems, let alone physics, from the perspective of process? After all, physics has been highly successful in its study of inanimate matter, in its use of mathematical language and structures to describe its behavior, and in elucidating the supposed general principles that underlie all of dynamics. Prior to the discovery of quantum mechanics, it would not have been given a second thought. However, hidden within physics’ success lies a host of assumptions that only began to be questioned in earnest over the past century. Physics took the easy path, studying relatively simple (or at worst complicated) systems, assumed to be isolated, closed, small, homogeneous (or at least not too diverse), stable, near equilibrium, non-contextual, having fixed components, linear dynamics, and being entirely reactive. Mathematics is well suited for the study of such systems, and the partnership between mathematics and physics has been remarkably fruitful. This success has been mutually reinforcing, but not always in the best ways. It has led to rigidity in viewpoints and some arrogance, especially when physics has sought to extend its scope outside of its usual domain.

Organisms behave wholly unlike inanimate matter. Organisms are fundamentally and necessarily open, acting, interacting, possessing multiple interacting subsystems, wildly diverse, and metastable; having components that are sometimes fungible, sometimes critical, but constantly in flux; having behaviors that are transient, often singular, recurrent but not periodic, generated or constructed rather than elicited, far from equilibrium, and contextual; and often engaging in nonlinear dynamics. Turning reductionism on its head, there have been attempts to apply concepts from the study of complex systems to the study of fundamental phenomena [19,20,21,22,23]. Experiments in psychology have demonstrated the existence of contextuality in a form familiar to quantum mechanics [24,25]. Indeed, there is now an extensive body of literature dealing with the application of quantum-mechanical formalisms to economics, psychology, and the social sciences [26,27,28,29,30]. The discoveries over the past century of deterministic chaos, non-equilibrium thermodynamics, nonlinear dynamics, fractal geometry, Levi and Pareto distributions, self-organization, self-organized criticality, the edge of chaos, emergence, adaptation, and resilience, as well as discoveries in collective intelligence and of the constructive nature of neural activity and human behavior, have all been powerful in motivating the study of complex systems, yet they still do not capture the essence of process, at least in the sense of Whitehead [19,20,21,22,23]. The persistence of old worldviews is not just a problem in physics but appears to plague many other disciplines as well [4,31,32]. Nevertheless, insights gained from the study of organisms and social science entities has kindled interest in an alternative worldview based upon the concept of process [33], and some have explored its application to physics, particularly to quantum mechanics [4,7,8,31].

The purpose of this paper is not to argue for Whitehead’s process theory [2] or to provide a detailed exposition of his ideas. His book, *Process and Reality*, is long, dense, and a daunting read. There have been many excellent summaries of his ideas [6,7,34], and readers who are interested in a more detailed examination of his writings are directed there. Likewise, this paper does not argue for the use of the process algebra (its application to NRQM has been described in a series of papers, starting with [35]), although the next section briefly presents one model that has been applied to NRQM, and readers who are interested in a deeper examination of its approach are directed to [3,35]. This section, loosely based on the presentation by Shimony [6], is meant to provide a basic conceptual background for the process algebra, and Appendix A provides some basic formal details of the process algebra itself.

The standard ontology that grounds most of our models is based upon a concept of *being*: entities *are*; they *persist* and *endure*; and we study equally enduring relationships between them. This ontology is founded upon the classical concept of object, inspired by our observations of inanimate matter [4,5]. A rock, for example, has a fixed set of constituents in a fixed arrangement, and its motion can be thought of as a continuous translation through space. An organism, on the other hand, has no truly fixed constituents. Its constituents are constantly in flux; its identity is determined by its history, by informational considerations. Watching an amoeba move is a profound experience. It does not simply translate; rather, it oozes in multiple directions, creating new cellular membrane along the propagating front and resorbing membrane along the receding front. It continually creates and destroys. Its motion is a sequence of becomings and fadings away.

Whitehead, in his ontology, proposed a radical shift in understanding, setting the concept of *becoming* as logically prior to *being*. This is a profound form of becoming, not merely marking the beginning and ending of some object but taking place *at every moment* in the history of an entity. An entity is viewed as a sequence of events, a sequence of successive becomings, linked together causally and informationally by process. The most primitive of these events, i.e., the basic facts of reality, he called *actual occasions*. These actual occasions come into being, persist just long enough to pass their information on to the next generation, and then fade away. Whitehead’s reality is what philosophers term a *compound present*, as opposed to a static block of enduring events, as is usually depicted in physics (a compromise is a growing block, in which the past endures while the future remains open until achieved) [5,36]. Actual occasions become actualized facts through what Whitehead termed *concrescence*.

Concrescence is a concept of fundamental importance in Whitehead’s metaphysics, referring to the process by which the plurality of potentiae becomes manifest in the actualization of the one. Simply put, a stochastic system realizes one of its possibilities. Following concrescence, an actual occasion becomes actualized or realized, becomes an objective fact, through what Whitehead termed *termination*. Whitehead does not separate out things from concrescences. There is no thing that undergoes concrescence; rather, it is a concrescence alone that is a thing. Entities are to be understood as processes and not objects.

An aspect of Whitehead’s conception of actual occasions that is fundamental to the process algebra approach is the idea that actual occasions must be treated as a complete whole. Any subdivision of an actual occasion into lesser parts can only be heuristic in nature, an outcome of our tendency to situate each occurrence of an actual occasion within some mathematical continuum. For Whitehead, such continua were not concretely real but rather reflected the set of potentiae available to an actual occasion, but not actual occasions themselves. Hansen describes this core of Whitehead’s idea as follows:

So, one central point in Whitehead’s system is that processes are themselves active-the temporal modalities are a function of the happening of processes themselves-the basic realities in the world pass from potentiality through actuality into pastness. Each process is a unit of becoming, and according to Whitehead it “becomes in solido”; it is not some temporally extended entity placed on an axis of time along which it could be played, like an audiotape, one stage or movement after the other. The idea is not that it is of zero extension like a point. It is the more radical idea that it is something different from extension—something out of which extension is *manifested*. Within this relational system, according to its properties, it may become meaningful to assign the process a finite extension. …However, this account of modality should not be taken as only monadic.

([31] pp. 153–154)

According to Whitehead, reality is discrete; nevertheless, continuity appears in the form of an abstract, conceptual, extensive continuum.

Actual entities atomize the extensive continuum. This continuum is in itself merely the potentiality for division; an actual entity effects this division. The objectification of the contemporary world merely expresses that world in terms of its potentiality for subdivision and in terms of the mutual perspectives which any such subdivision will bring into real effectiveness…With the becoming of any actual entity what was previously potential in the space-time continuum is now the primary real phase in something actual. For each process of concrescence a regional standpoint in the world, defining a limited potentiality for objectifications, has been adopted. In the mere extensive continuum there is no principle to determine what regional quanta shall be atomized, so as to form the real perspective standpoint for the primary data constituting the basic phase in the concrescence of an actual entity.

([2] p. 104)

Actual occasions form as complete wholes, but we can imagine them subdivided because we project this extensive continuum onto them, even though the latter does not represent actual divisions.

Whitehead wrote,

Our direct perception of the contemporary world is thus reduced to extension, defining (i) our own geometrical perspectives, and (ii) possibilities of mutual pespectives for other contemporary entities *inter se*, and (iii) possibilities of division. These possibilities of division constitute the external world a continuum. For a continum is divisible; so far as the contemporary world is divided by actual occasions, it is not a continuum, but is atomic. Thus the contemporary world is perceived with its potentiality for extensive division, and not in its actual atomic division.

([2] p. 96)

For Whitehead, the concrete actualities that form our reality, which are actual occasions, occur discretely and with finite extension in both time and space. Nonetheless, the actual when and where attributed to such occasions need not arise from a discrete set of possibilities. These values are taken from an abstract set of potentiae, of possibilities, and this set may well be continuous in nature. These values may be derived from a continuous set of real numbers, for example. However, it is important to distinguish between the concrete reality of actual occasions and the abstract ‘reality’ of these values that comprise the *extensive continuum*. Making this abstract extensive continuum concrete is an example of the Fallacy of Misplaced Concreteness.

Whitehead makes this clear in his understanding of becoming:

Finally, the extensive continuity of the physical universe has usually been construed to mean that there is a continuity of becoming. But if we admit that ‘something becomes’, it is easy, by employing Zeno’s method, to prove that there can be no continuity of becoming. There is a becoming of continuity, but no continuity of becoming. The actual occasions are the creatures which become, and they constitute a continuously extensive world. In other words, extensiveness becomes, but ‘becoming’ is not itself extensive.

([2] p. 53)

The concept of ‘event’ plays a fundamental role in most physical theories. Whitehead defines an event as follows:

The term ‘event’ is used in a more general sense. An event is a nexus of actual occasions inter-related in some determinate fashion in some extensive quantum: it is either a nexus in its formal completeness, or it is an objectified nexus. One actual occasion is a limiting type of event. The most general sense of the meaning of change is ‘the differences between actual occasions in one event’.

([2] pp. 124–125)

Epperson [7] has argued that Whitehead’s philosophy is consistent with the group of interpretations of quantum mechanics called ‘event interpretations’.

In Whitehead’s system, *processes* express dynamics; actual occasions *do not do anything*. They do not move, they do not change state, they do not interact. Processes do these things. Actual occasions are more akin to the entries on a scratchpad or the symbols acted upon by a Turing machine. Echoing Aristotle, Whitehead [2], and Arthur [18], *nothing happens at a point*. Actual occasions have extension in both space and time. Their temporal extension constitutes an interval of time, termed a *duration*, rather than a point or instant of time, and their spatial extension constitutes a *region* of space, not a *point*. An instant of time simply marks the end of the concrescence (termination) of one actual occasion and the beginning of the concrescence of another. Thus, each actual occasion [n] is associated with one instant of time (*initiation*), $i_{n}$, which marks the onset of its concrescence, and a second instant (*termination*), $t_{n}$, which marks the end of its termination. If $i_{n},t_{n}$ are assigned real values, then the *duration* $d_{n}$ of [n] is given as $d_{n}=t_{n}-i_{n}$. Actual occasions do not occur *in* time and space; rather, they *constitute* time and space.

The question as to whether processes move is open. The ‘modalities are not really situated in space and time at all, but in the concrete processes whose web of relations gives rise to space and time’ [31]. Gisin [37,38] posited a similar speculation about the nature of entanglement. In Whitehead’s view, space and time are not pre-existing ‘containers’ within which events play out, but, rather, space and time are themselves emergent constructs, relations arising through the generation of events by the actions of processes. Rovelli [39] too has emphasized a view of space and time as relations and not physical entities.

The ‘tossed coin metaphor’ [4] was introduced to help stimulate thinking about some of these ideas but in a more accessible classical setting. One imagines a device for chaotically propelling a fair coin into a vertical trajectory, below which is a motion-dampening platform that can be placed at different heights and at different angles. A button initiates the toss, and, when the coin reaches the platform, it rapidly dissipates its energy, coming to rest in either a (*H*)eads-up or (*T*)ails-up position. This is indicated on a display. Note that the state *H* or *T* is a state of the display and exists only when the coin lies at rest on the platform. During the time between the pushing of the button and the activation of the display, the coin is in motion and thus is never in a state of *H* or *T*. Neither *H* nor *T* is a state of the coin but only of the display. Now, suppose that the entire apparatus is encased in an opaque box, with only the button and the display visible. One presses the button, waits, and an *H* or *T* is displayed. These are the actual occasions of this apparatus—the determinate facts of the apparatus. The entire apparatus is a process that generates *H* or *T*.

Internally, the tossed coin is a process that, together with the surrounding apparatus, generates these facts. It is not a possessor of these facts. A state of the coin might be provided by the motion of a normal to the surface bearing the head, together with any angular momentum having this normal as its axis. Such states have no direct relationship with the appearance of *H* or *T*. There is neither counterfactual determinism nor microscopic realism [40] with respect to *H* or *T*, yet the coin is perfectly real, as is the apparatus. One might say, ‘out of sight, out of mind, but not out of reality’. Sealed from observation, the internal components provide a collection of hidden processes having their own hidden variables, but none correspond to *H* or *T*. It is the apparatus as a whole that generates a single actual occasion once the button is pressed, and this must be treated as a whole; there is no decomposing the process of the concrescence of these actual occasions without destroying the apparatus. The actual occasions of the apparatus, represented by *H* and *T*, are not instantaneous but have a duration, extending from the pressing of the button to the appearance of a value, *H* or *T*, and thus an extension in time and an extension in space as well—that of the apparatus.

At the level of the measurement apparatus, it is exact to describe it via a wave function of the form $\frac{1}{\sqrt{2}}\left|H>+\frac{1}{\sqrt{2}}\right|T>$ since, indeed, *H* and *T* will each appear with probability 1/2. However, there is no ‘collapse’ of this wave function upon pressing the button. This wave function is purely epistemological. It reflects our knowledge if all that we have access to is the whole apparatus. It certainly does not describe what transpires within the apparatus once the button has been pressed. It is not ontological. There is no magic in the appearance of *H* or *T*, although, if we cannot open the apparatus, we will not know this. The wave function provides a complete description of the apparatus only if we are only interested in the appearance of either *H* or *T*. It is not if we ask some other questions.

Limitations in our ability to observe, or to acquire knowledge about something, do not necessarily imply the absence of that something. In [41], I suggested that we define something as being ‘real’ if it can be demonstrated that that something makes a difference. Here, the tossed coin is real, even if an external observer cannot know of its existence. They may not know the ‘what’ of that internal something, but they can assert that there is a something present. Likewise, an actual occasion must be treated as a whole, but the process of concrescence, which makes an actual occasion actual and determinate, must be presumed to be real. We may never know exactly what it is, but we can create heuristic models to aid in our predictions or understanding, so long as we do not fall into the trap of the Fallacy of Misplaced Concreteness, and we assume that our model entities are somehow real.

It is important to always keep in mind the distinction between ontology (what is) and epistemology (what is known). In the process algebra, this is expressed in part by the intrinsic (ontology) and extrinsic (epistemology) distinction. The tossed coin metaphor tries to make this point, distinguishing between what is—the tossed coin and the measuring apparatus—and what is observed—namely, the registration of an *H* or *T*. Relativity showed that observations of a system by an observer moving relative to it may confuse cause and effect. It is possible for such an observer to observe the appearance of an effect prior to the appearance of its cause [42], but this does not alter the actuality of those events. Only simultaneous events could possibly be directly observed, while all other events require signals, and, if signals are involved, then cause and effect can become confused or indefinite. Robb [43] pointed out that the only truly simultaneous events are those that occupy exactly the same spacetime location. Epistemology does not always respect ontology. This makes the study of cause and effect challenging. In the process algebra approach, causality refers to what the process does and not what an observer might think it does.

In regard to causality, I prefer to follow Bunge [44] and to think in terms of mechanisms of determination of events and, in the context of process, to think of influences on such determination, rather than simple causes, since, invariably, there is no simple linear relation ‘cause to effect’ but rather a complex interaction among multiple prior actual occasions in the determination of any nascent actual occasion. Whitehead’s views on causality are deep and complex, and their discussion would take us far from the main subject of this paper. Nevertheless, a few comments are in order.

Whitehead does not describe a linear relationship between cause and effect. He writes,

In the becoming of an actual entity, the *potential* unity of many entities—actual and non-actual—acquires the *real* unity of the one actual entity; so that the actual entity is the real concrescence of many potentials.

((SIC) [2] p. 33)

Every actual occasion arises through an act of *prehension* by the nascent actual occasion of its prior actual occasions. Whitehead’s Theory of Prehensions occupies 100 pages and is quite complex. In the process algebra, Whitehead’s concepts of concrescence and prehension are subsumed within the concept of information propagation and its representation in some particular model.

Whitehead writes,

Every condition to which the process of becoming conforms in any particular instance has its reason *either* in the character of some actual entity in the actual world of that concrescence, *or* in the character of the subject which is in process of concrescence. This category of explanation is termed the ‘ontological principle’.

((SIC) [2] p. 36)

The concrescence of an actual occasion involves an interplay between a collection of prior actual occasions and a nascent actual occasion, taking potentiality and making it determinate. The contribution of the nascent actual occasion to its own concrescence is what is termed prehension. The study of prehension is beyond the scope of this paper.

Prior to the concrescence, there is only potentiality, or indeterminateness, and Whitehead wrote that ‘…This indetermination, rendered determinate in the real concrescence, is the meaning of ‘potentiality’. It is a *conditioned* determination, and is therefore called a ‘*real* potentiality’’ (SIC) [2] p. 34. In stating this, I believe that Whitehead is expressing Bunge’s concept of stochastic determination. This is not the classical deterministic determination, again using the terminology of Bunge.

Whitehead wrote,

There is a prevalent misconception that ‘becoming’ involves the notion of a unique seriality for its advance into novelty. This is the classic notion of ‘time’, which philosophy took over from common sense. Mankind made an unfortunate generalization from its experiences of enduring objects. Recently physical science has abandoned this notion. Accordingly we should now purge cosmology of a point of view which it ought never to have adopted as an ultimate metaphysical principle. In these lectures the term ‘creative advance’ is not to be construed in the sense of a uniquely serial advance.

([2] p. 52)

Whitehead is denying the preeminence of a deterministic seriality in the unfolding of actual occasions and in the process of concrescence. Nevertheless, there still exist temporal distinctions since, following an instance of concrescence, the prior actual occasions fade away, and only the nascent actual occasions persist into the next generation, eventually to fade away themselves. From this, one may derive an ontological temporal order at the level of actualized actual occasions. There is no need for a strict ‘temporal’ ordering that includes information propagation within the act of concrescence, since any such division is purely heuristic and thus arbitrary. The opacity of actual occasions might open the door to some kind of causal indefiniteness in Whitehead’s thinking, but this is a matter for further examination.

Causal indefiniteness and questions of temporal reversibility are subjects of interest in quantum mechanics [45]. In both Whitehead and the process algebra, there may be causal indefiniteness in the sense that there are often many ‘causes’ contributing to the generation of any actual occasion, not *a* cause. Moreover, the potential participation of the nascent actual occasion via prehension in its own concrescence makes statements about causation less clear. However, the process of concrescence is generally considered to be unobservable, and, at the level of the actualized actual occasions themselves, there is a strict asymmetry marked by the order of their becoming.

## 3. The Process Algebra and Quantum Mechanics

As discussed in [35], the process algebra was introduced to address questions in quantum foundations. In particular, it was introduced to provide an example of a realist, local, contextual model of NRQM and thus to demonstrate that non-locality is not a necessary feature of NRQM. Although the process algebra could be used for calculations, its main purpose was to address various philosophical issues related to interpretations of quantum mechanics. An outline of the process algebra is provided in Appendix A. For illustrative purposes, consider one model presented in [35], the path integral model. Since this is a model of NRQM, we may take as the interpretation space $R^{4}$ with the usual separation into one time dimension and three spatial dimensions, together with the usual metric $ds^{2}=dt^{2}+dx^{2}$. Assume that we are dealing with a single quantum system described by a standard Schrödinger equation of the form

(1) $$i\hbar \frac{\partial \Psi }{\partial t}=-\frac{\hbar^{2}}{2m}\nabla^{2}\Psi +V\Psi$$

for some time-independent potential function *V*.

Assume that there exists a Feynman propagator $K(t_{1},x_{1}:t_{2},x_{2})$ for a path integral version of this problem. To ensure local, causal propagation, we assume that $K(t_{1},x_{1}:t_{2},x_{2})=0$ whenever $|x_{2}-x_{1}|/\left(t_{2}-t_{1}\right)>c$, the speed of light in vacuo. The actual occasions of this model have the form $\left[n\right]<m_{n},\phi_{n};p_{n},\Gamma_{n}>\left\{G_{n}\right\}$, where *n* is a 4-tuple of integers; $n=(n_{t},n_{1},n_{2},n_{3})$, $m_{n}$ is a lattice embedding of *n* into $R^{4}$ with scale $l_{T}$ for the time dimension and $l_{S}$ for the three spatial dimensions (for example, $\left(n_{t},n_{1},n_{2},n_{3}\right)\to \left(n_{t}l_{T},n_{1}l_{S},n_{2}l_{S},n_{3}l_{S}\right))$; and $\psi_{n}=\Gamma_{n}\phi_{l_{T},l_{S}}\left[m_{n}\right]$, where $\phi_{l_{T},l_{S}}\left[m_{n}\right]$ is shorthand for the four-dimensional sinc wavelet (sinc(x)=sin(x)/x) centered on the point $m_{n}$ and having wavelengths $l_{T},l_{S}$, respectively [46]. This is the *local* interpreted wave function. The property vector $p_{n}$ will depend upon the preparation and, in general, will match the set of intrinsic properties associated with its generating process. This ensures that the propagation of information from any occasion will be linked precisely to the process that generated it. In this particular example, we are considering the basic time-independent Hamiltonian situation, so $p_{n}$ can be taken as simply the energy eigenvalue. We ignore $\left\{G_{n}\right\}$ for this brief example. Let $I_{k}$ denote the collection of actual occasions forming generation *k*. Assume that this is the prior generation. The actual occasions of the nascent generation $I_{k+1}$ are generated by means of a two-player combinatorial game, whose details do not matter as the use of the game is purely heuristic. The game randomly places a nascent actual occasion at some empty site on the sub-lattice $\left\{\left(k+1,n_{1},n_{2},n_{3}\right)\right\}$ and propagates information locally and causally from the prior actual occasions to the nascent actual occasion $n^{\prime }$ via

(2) $$\Gamma_{n^{\prime }}=\sum_{n\in I_{k}}K\left(m_{n}:m_{n^{\prime }}\right)\Gamma_{n}$$

Here, locally means that any information can propagate no further than the distance that light could propagate from an actual occasion during the duration of a generation, while causally means that the speed attributed to such propagation must not exceed that of light (in vacuo), and, if interpreted via a causal manifold, then any nascent actual occasion must lie in the forward light cone of any prior actual occasion that is propagating information to it. Information here is meant in a very concrete sense as meaning laden information, a concrete datum, most often propagated by means of some signal and sign (at least between processes, although not necessarily within a single process). This is not information in the sense of quantum information, which has an altogether different character, more closely linked to operators, circuits, probabilities, and extensions of Shannon type information theory in which information relates to losses of uncertainty.

The *global* interpreted wave function $\Psi^{\Pi }\left(t,x\right)$ on $R^{4}$ is obtained via four-dimensional sinc interpolation as

(3) $$\Psi^{\Pi }\left(t,x\right)=\sum_{[n]\in I}\Gamma_{n}\phi_{l_{T},l_{S}}\left[m_{n}\right]$$

where *I* is the current causal tapestry, the union of all previously created generations.

Let Ψ(t,x) denote the wave function obtained directly from the Schrödinger equation. In [35], it was shown that, as the number of actual occasions grows (so that the amount of information regarding the wave function increases), the discrepancy between the Schrödinger wave function and the global interpreted wave function decreases. The actual discrepancy also depends on the scales $l_{T},l_{S}$, which set the maximum possible energy of the quantum system under investigation. The global interpreted wave function possesses a natural ultraviolet cutoff due to the finiteness of these scales. Assuming an infinite number of actual occasions, so that the lattice is saturated, and scales set at Planck time and Planck length, the discrepancy [46] is

$$|\Psi \left(t,x\right)-\Psi^{\Pi }\left(t,x\right)|<10^{-97}\left(m^{-3}\right)$$

so that the two functions are virtually identical. With 300,000 actual occasions per generation, the discrepancy can be bounded by as little as $10^{-27}$ m^−3^, which is still well outside conceivable observational limits. There is no natural ultraviolet cutoff in the Schrödigner formulation, so that there will always be a slight discrepancy, but this will vanish in the limit as $l_{T},l_{S}\to 0$. In general, the number *N* of actual occasions created in a generation will be a function of the energy, and it is assumed sufficient to ensure that the temporal density matches or exceeds the Beurling density [47] associated with the wave function (this is necessary to ensure that the interpolated wave function will have a bandwidth equal to or greater than the Schrödinger wave function).

Since the details of the concrescence of an actual occasion are unknowable within Whitehead’s theory, any model of concrescence is necessarily purely heuristic. An important concept in the process algebra is *epistemological equivalence*, which holds that two process models are epistemologically equivalent if they produce the same global interpreted function over any causal tapestry. In the context of NRQM, we are thus only interested in epistemologically equivalent models that give rise to the same NRQM wave function. The details concerning how concrescence is carried out within the model are of little concern and may be chosen for computational or conceptual convenience. Only the actual occasions themselves, and their local interpretations, have any importance. The situation resembles that of gauge freedom in classical electrodynamics, where a particular gauge may be chosen for convenience, since it is only the resulting field values that possess any significance.

There is a measurement theory associated with the process algebra, but it is not relevant to the subject matter of this paper, and the reader is referred to [3,5,35] for further information. In addition, probabilities associated with the process algebra arise in two forms. First of all, there is the question of the selection of the individual actions that constitute the concrescence of actual occasions. The adoption of a combinatorial game-theoretic model of these process actions brings with it the notion of non-determinism as a mechanism of determination. Non-determinism is a concept from computer science and computability theory, as well as combinatorial game theory, and explains the determination of a particular action as a consequence of a *choice*. This choice is *not* determined by an a priori probability (although one could apply a uniform distribution as a default) but is usually conditioned by the prior and current context. Probabilities may be ascribed to action selection by measuring frequencies of action choices, assuming that the context remains fixed, but, in most situations, the context will be constantly changing and so any emergent probability will be non-stationary, if it exists at all. There is no reason that these frequencies should converge over time to give consistent values that can be interpreted as probabilities in general. The second type of probability relates to the probabilities of measurements. These probabilities are emergent and dependent upon the coupling between the system and measurement process (based on a concept from Trofimova [20]). These probabilities are dependent on actual occasions and primarily on the information associated with actual occasions. This information may also be conditioned by the context and by interactions. In the path integral model, information includes values attributed to a wave function and so probabilities may be determined from these values.

In the process algebra approach to NRQM, it is possible to claim that the wave function associated with a single quantum system is both ontological and epistemological—that is, it both describes the system *as it is* and it may also be used for calculating probabilities of potential measurements. The action of process generates a succession of generations of actual occasions, forming a discrete causal tapestry, which can be embedded via the interpretation into $R^{4}$. Recall that a sinc wavelet has the property that its value at its center is 1, so that the value of the local interpreted wave function $\psi_{n}=\Gamma_{n}\phi_{l_{T},l_{S}}\left[m_{n}\right]$ evaluated at the embedding point $m_{n}$ is just $\Gamma_{n}$. The global interpreted wave function is thus a discrete sampling of the continuous Schrödinger wave function, and, if the scale at which the actual occasions is Planck scale, then, at macroscopic scales, this discrete version will effectively appear like the continuous version. However, the values of the global interpreted wave function correspond to actualities at events represented by the causal tapestry, not simply potentialities. Moreover, note that uncertainty relations are guaranteed by virtue of fundamental results in interpolation theory [46].

From the process algebra perspective, a superposition $\Psi \left(x\right)=\sum_{i}w_{i}\Psi_{i}\left(x\right)$, is generated by a global process Π that is a process algebra sum of subprocesses $\Pi_{i}$, one for each eigenstate, $\Psi_{i}\left(x\right)$, so we have $\Pi ={⊕}_{i}w_{i}\Pi_{i}$. Each subprocess $\Pi_{i}$ will generate a distinct collection of actual occasions $I_{k}^{i}$ at each generation *k*, forming the causal tapestry $I^{i}$. These collections are disjoint, so that no actual occasion can be viewed as existing in or manifesting more than a single eigenstate. The global interpreted wave function is a sum, an intertwining, of global interpreted wave functions—one for each sum process. Formally,

(4) $$\Psi^{\Pi }\left(x\right)=\sum_{i}\Psi^{\Pi_{i}}\left(x\right)=\sum_{i}\sum_{n\in I^{i}}\Gamma_{n}\phi_{l_{T},l_{S}}\left[m_{n}\right]$$

The actual occasions corresponding to the distinct states all contribute to the resulting wave function, but each only to a single eigenstate, and the probabilities are determined in the usual manner, so that $Prob_{i}={\left|w_{i}\right|}^{2}$.

Suppose, however, that we have two processes $\Pi^{1},\Pi^{2}$ and we consider the product process $\Pi^{1}⊗\Pi^{2}$. The actual occasions of the two processes must lie in disjoint sets $I^{1},I^{2}$, respectively, and one can define an ontological wave function for the product process in the usual manner:

(5) $$\Psi^{\Pi^{1}⊗\Pi^{2}}=\sum_{n\in I^{1}}\Gamma_{n}\phi_{l_{T},l_{S}}\left[m_{n}\right]+\sum_{m\in I^{2}}\Gamma_{n}\phi_{l_{T},l_{S}}\left[m_{n}\right]$$

This wave function, however, is *not* the product of the wave functions of the two processes but instead is the coproduct of the wave functions. To define the product, we must first move to a configuration space. The global process generates pairs of informons $\left(n^{1},n^{2}\right)$ at each step to construct a generation $I_{k}$. There will be a generation $I_{k}^{1}$ for subprocess $\Pi^{1}$ and $I_{k}^{2}$ for subprocess $\Pi^{2}$. However, $I_{k}\neq I_{k}^{1}\times I_{k}^{2}$; rather, we have only that $I_{k}\subset I_{k}^{1}\times I_{k}^{2}$. Thus, the global interpreted wave function of the product will not be the product of the global interpreted wave functions of the components,

(6) $$\Psi^{\Pi^{1}⊗\Pi^{2}}\left(x,y\right)\neq \Psi^{\Pi^{1}}\left(x\right)\times \Psi^{\Pi^{2}}\left(y\right)$$

The problem is that, in general, the global process will not be ergodic over the configuration space; so, in order to create the usual Schrödinger product wave function, it is necessary to shift from the causal tapestry to what is called the process-covering space, essentially running the process again and again and keeping track of the generated causal tapestries, followed by taking the graph union of them all. This is no longer an ontological entity but merely an epistemological entity, useful for calculations but not for describing a particular generated reality. Thus, while the coproduct of wave functions may be ontological, the product of wave functions is merely epistemological. I suggested in [5,35] that some of the confusion in interpreting quantum mechanics is due to the definition of the wave functions, which uses a simple function sum or product to construct more complicated wave functions, thus conflating different ontological states and blurring the distinction between ontology and epistemology. The process algebra formalism keeps track of how processes are constructed and interact, maintaining ontological and epistemological distinctions.

This is particularly true of entanglement situations, such as when one has two particles that can appear in mutually exclusive states a,b and one forms the entangled wave function $\Psi =\Psi_{a}^{1}\Psi_{b}^{2}+\Psi_{b}^{1}\Psi_{a}^{2}$. This standard representation as wave functions makes it appear as though this pair of particles is somehow in some ontological superposition of states all the time. The process algebra formulation takes the form

(7) $$\Pi =\left(\Pi_{a}^{1}⊗\Pi_{b}^{2}\right)⊕\left(\Pi_{b}^{1}⊗\Pi_{a}^{2}\right)$$

In this formulation, one sees that the entanglement situation consists of a pair of subprocesses, each consisting of a product of two lesser subprocesses. The first-order subprocesses generate a pair of actual occasions at each step, one from process $\Pi^{1}$ and the other from process $\Pi^{2}$. Their coupling into a single process maintains the correlations between the observed measurements, in spite of information being propagated locally and causally. The resulting probability distribution violates the factorizability condition described by Shimony [6], demonstrating the possibility of contextuality and locality, although it is important to remember that actual occasions are not hidden variables. They are not necessarily observable, although they do play a role in interactions among processes. They are, nevertheless, events in Whitehead’s sense.

There is no ‘spooky action at a distance’ or ‘passion at a distance’ [6] in the process algebra. Instead, process serves as a common cause that generates pairs of correlated actual occasions [48]. If process $\Pi_{a}^{1}⊗\Pi_{b}^{2}$ is acting, then only actual occasions of $\Pi_{a}^{1}$ and of $\Pi_{b}^{2}$ are being generated, and this occurs causally and local to each individual process. The same holds for the process $\Pi_{b}^{1}⊗\Pi_{a}^{2}$. These actions do not take place *in* space and time because these actions *generate* space and time elements corresponding to the actual occasions being generated.

The process algebra is, in many respects, a pre-quantum theory (see [8] for other examples). In [35], it was shown that it is possible to develop an operator-based theory of process, but, in so doing, one must give up ontology and focus instead on epistemology. This is currently an underdeveloped area of research, but it raises the possibility of developing a more direct link between the operator formalism of quantum mechanics and the process algebra. In situations in which the fundamental actual occasions are not observable, an operator approach may be necessary.

The acquisition of measurements requires an interaction between the system process of interest and a specific measurement apparatus process. The actual occasions of the system process may not be directly observable, and only the actual occasions of the system–measurement process may be observed. This will give rise to a different process algebra model, having a distinct description and different operator structure. Recall the tossed coin metaphor. It is perfectly reasonable to describe the probability structure of the measurement apparatus by a wave function such as $\Psi =\frac{1}{\sqrt{2}}\left|H>+\frac{1}{\sqrt{2}}\right|T>$. This is complete as far as determining the probabilities of measurements is concerned (and is time-independent and thus effectively time-reversible), but it is incomplete if one wishes to describe what takes place within the measurement apparatus. For this, a different set of processes and actual occasions is required, which are not time-symmetric. The use of an operator formalism might result in causal indefiniteness of the type observed in quantum mechanics and quantum information, but, at the level of the actual occasions themselves, causal indefiniteness would seem unlikely. It certainly might be a feature of concrescence, but this would then appear in individual models, since concrescence itself is held to be opaque.

Aside from possessing natural infrared and ultraviolet cutoffs, the discreteness and finiteness of the process algebra ensure that the propagation procedure always terminates, thus always generating a finite collection of actual occasions. Thus, a global interpretation wave function can be generated for any initial and boundary conditions. This is not always guaranteed in the continuous case. Whether the resulting wave function is consistent with the physical situation or closely approximates the Schödinger wave function is a matter of verification. That this function is created from generation to generation implies that it might be better suited to situations in which there is considerable temporal variability or unpredictability in the Hamiltonian. This would be true of open systems and systems interacting with an active environment, such as when dealing with complex adaptive systems. The approach needs to be applied to a much broader range of problems to evaluate whether or not this proves to be the case.

## 4. The Process Algebra View of Time

From the viewpoint of process, time and space may not be equivalent but they are inextricably intertwined; thus, before addressing the issue of space, it is necessary to first briefly review how time is generated by process. There are many different conceptions of time [18,38,39,49,50,51,52,53], including that time does not exist [39]. Arthur [18] proposes a meticulous argument for the reality of temporal flow, and others have presented similar cases [38,50]. Arthur proposed the idea of local temporal flows, and similar ideas of fragmented time have been put forward by several authors within the setting of tensed theories in philosophy [54,55]. Even Rovelli, who denies that time exists, nevertheless argues for a fundamental role for becoming, or happening [39]. Thus, what I describe here, while not mainstream, is not without precedent.

A central feature of the process algebra approach is the separation of ontology and epistemology. In the process algebra approach, ontology—a description of what was or is—is provided by a causal tapestry, a history of the generation of actual occasions by some process (see Appendix A for the definition of a causal tapestry). This formal structure assumes only the existence of an ordered graphical relational structure associating these actual occasions to one another. The details of this structure are the focus of this paper and follow below. The epistemology is provided by what I have termed the interpretation, which provides an embedding of these actual occasions into a more familiar mathematical structure, such as a manifold, together with a function formed from the information content of these actual occasions—not by asymptotically scaling the actual occasions to an ever smaller size but rather through an interpolation procedure (based on an idea from Kempf [56,57]). The concrete atomistic actuality always remains discrete and finite, while interpolation provides the bridge to an extensive continuum. The term interpretation is used precisely because it is a choice of some observer as to how they wish to model the ontology—the value of an interpretation thus depends upon what features of the ontology the observer wishes to emphasize and the goodness of the fit between the two. It is intended to reflect the fact that, at the most fundamental level, reality cannot be directly perceived and we are left with how our brains process sensory information about the world—processing that has been hardwired through evolution to provide an effective means to interact with our meso-scale reality.

In Whitehead’s process theory, becoming or happening is considered to be an ontological primitive. Events happen. From the process viewpoint, happenings do *not* happen *in time*. Happenings happen, giving rise to temporal distinctions. A temporal distinction is a relation the marks these happenings, that marks change. Temporal distinctions are epistemological relations between entities or events that are related ontologically by a happening. Temporal distinctions are what we colloquially refer to as time. There are many different temporal distinctions. In physics, and commonly in philosophy as well, it is assumed that these various temporal distinctions describe one and the same time. This might be true for ontology, but it does not seem necessary, a priori, for this to be true when time is considered from an epistemological viewpoint.

McTaggart’s [36] famous A, B, and C series represent three temporal distinctions, while clock time and spacetime are two others. The basic ordering of happenings is called *ontological time*. The ticking of a clock with which we associate events provides another set of temporal distinctions that I call *measurement time*, since it relies upon an act of measurement by means of some measurement process. Closely related is *metric time*, when each related pair in a temporal distinction is associated with a numerical value from whose numerical order relation the temporal order can be derived.

The notion that ontological and measurement time might not be the same is discussed in [5]. Briefly, one might argue that one merely needs a sufficiently sensitive clock. However, a clock must be a process itself, and the tickings of the clock become the actual occasions of some measurement system. Let the frequency of the clock be ω. In the process algebra formulation, let the interpretation of these tickings be a one-dimensional real manifold, so there will be an embedding *e* of the tickings of the clock into this manifold. The position of the clock can be given by some parameter, such as an angle θ, which serves as information for each actual occasion. We use sinc interpolation [46] to generate the interpretation function (see Appendix A), which takes the form

(8) $$f\left(t\right)=\sum_{n}\theta_{n}sinc(2\pi \sigma \left(t-e\left(\left[n\right]\right)\right)$$

Now, the frequency of the clock is ω, so, according to sinc interpolation theory, the sampling frequency σ must be at least 2ω—that is, either the ticks must occur at twice the frequency that the ticks occur, which is a contradiction, or the actual occasions of the clock must be generated at at least twice the frequency of the ticks being recorded. It is not possible for the clock to measure the timing of its own actual occasions, nor can it measure the timing for any higher-frequency events. The idea of an ultraviolet cutoff suggests that there are physical limits to the maximum frequency of its phase. A fundamental particle at its ultraviolet cutoff would need to generate actual occasions at twice this maximal frequency and thus cannot have measurable or metric temporal distinctions, although it would still have ontological temporal distinctions.

The use of sinc interpolation in the example above might lead one to conclude that happenings at the level of actual occasions must occur with some kind of regular periodicity, but this is not necessary. It has been shown that sinc interpolation can be carried out with non-uniform sample timing [58], so long as the density of sample points equals or exceeds the Beurling density [47]. In fact, new methods have been developed to deal with interpolation with non-uniform sampling more generally [46], but sinc interpolation is shown here simply because it is easier to understand.

Arthur [18] points out that events happen when they happen, and not at any other time. This becoming is difficult to depict mathematically, as mathematics is better suited to dealing with the *history* of a succession of happenings, rather than the happenings themselves. In the process algebra, history is provided by a causal tapestry. If the causal tapestry is interpreted (see Appendix A) as a physical spacetime, then a common interpretation structure would be a causal manifold [59], into which the causal tapestry is embedded. Different interpretations would involve different interpretation structures, and different observers would result in different embeddings into these structures. An important point to remember is that the causal tapestry describes ontology, while the interpretation reflects epistemology.

As Whitehead emphasized, the opacity of concresence to observation leaves us free to heuristically imagine internal states and actions, so long as, in the end, they yield the same epistemological facts (here, this means giving rise to the same interpolated functions, called *epistemological equivalence* in [35]). In the process algebra models studied to date [35], a process is assumed to create a set of actual occasions in discrete generations, and there will usually be many actual occasions created in each generation. Information only propagates causally (i.e., always at no more than the speed of light) and only among the actual occasions that comprise the immediate prior generation and those being created in the nascent generation. In the formal language of the process algebra, given some causal tapestry C and interpretation structure M, there will be an embedding e:C→M and a global interpreted function $\Psi \left(x\right)=\sum_{[n]\in C}\Gamma_{n}g\left(x,e\left(\left[n\right]\right)\right)$, where *g* is an interpolation kernel. Interpolation links the process-level phenomenology to what is usually described in our standard models. The propagation of information from prior to nascent actual occasions leading to the generation of each $\Gamma_{n}$ provides the basis for the causal structure of the causal tapestry and the source of its temporal distinctions. Given two actual occasions, [m],[n], we say that [m]<[n] or [m]→[n] if, in generating these informons, the process propagates information from [m] to [n]. The process order represents causal information flow. It does not reflect the order of happening (which may be synchronous or asynchronous) between actual occasions within a generation, since no information is propagated between actual occasions within a generation. It does not provide the most fine-scaled set of temporal distinctions, which can only be hypothesized and not observed. I refer to this as the *information causal order*, or IC order. The IC order or graph structure represents the history of the actual occasions generated by a process. It represents actualities and not potentialities. Note that, since actual occasions possess temporal extension in the form of durations, it may be more accurate to use interval orders rather than point-based orders [60].

**Figure 1:**
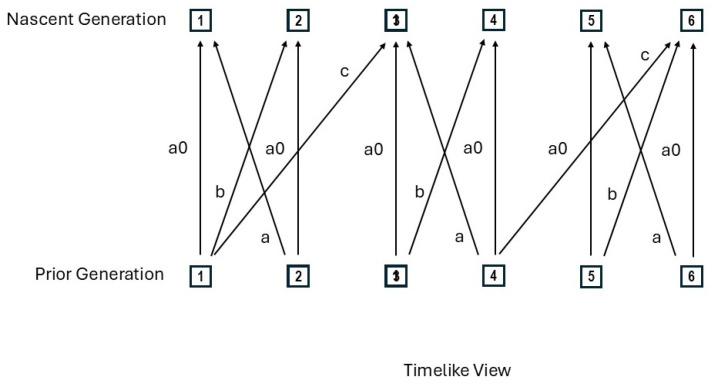
Timelike view. This figure depicts the temporal distinctions between a prior and a nascent generation. Each arrow represents the propagation of information from a prior to a nascent actual occasion, labeled by the action undertaken to do so. The actions are relative, not absolute; hence, they may be the same for different prior actual occasions. a0 is the unique action that defines positions, and the numbering is used to denote positions, not identities of actual occasions.

Thus, to each pair of generations, $I_{n}$ (prior), $I_{n+1}$ (nascent), one can associate a bipartite graph whose vertices are actual occasions in their respective generations and edges if information propagates from the actual occasion that is the initial vertex to the actual occasion that is the final vertex. Since no information propagates among the actual occasions within a generation, each generation contributes an anti-chain to the order. Thus, the IC order structure can be represented as a graph (IC graph) of the form

(9) $$\cdots ⊕G\left(I_{i},I_{i+1}\right)⊕G\left(I_{i+1},I_{i+2}\right)⊕G\left(I_{i+2},I_{i+3}\right)⊕\cdots$$

where each $I_{i}$ is an anti-chain of actual occasions forming a single generation, and each $G(I_{i},I_{i+1})$ is a directed bipartite graph with initial vertices in $I_{i}$ and terminal vertices in $I_{i+1}$, representing information flows; ⊕ is a graph sum. Figure 1, which appears in Section 6, depicts one such bipartite graph.

Each $G(I_{i},I_{i+1})$ represents a compound present, while the entire graph represents the history of the process. Each temporal ordering associated with a single process is purely local, an example of a local becoming, as described by Arthur [18].

While processes have beginnings and endings themselves, the beginning may lie in the indefinite past [61], so the order might resemble

(10) $$\cdots I_{-n}I_{-n+1}I_{-n+2}\cdots I_{-1}I_{0}I_{1}I_{2}I_{3}\cdots I_{m}\cdots$$

## 5. The Process Algebra View of Space

Let us now consider how space might be viewed from a process perspective. I do not review the myriad conceptions of space that have been put forward over the centuries, although there have been many that contribute to the process conceptions presented here [2,18,43,62,63,64]. There are two notable works that presage the approach to be presented here. Robb [43] developed his optical theory of space and time based on the idea of an ordering of events as a result of light signals being sent between them. From 21 postulates and 206 theorems, he was able to develop a four-dimensional Minkowski space structure with its usual pseudometric. Robb’s theory is specifically directed towards the development of a geometric, physical spacetime but does highlight how a spacetime structure can emerge from the propagation of signals. Whitehead also developed a theory of space in his chapter on the theory of extension [2]. Whitehead initially presented a fairly general theory but then focused on developing a model of a geometric, physical spacetime. Early on, though, he pointed out a distinction that is similar to that being advocated for here. He stated, ‘There are two distinct ways of ‘dividing’ the satisfaction of an actual entity into component feelings, genetically and coordinately. Genetic division is division of the concrescence; coordinate division is division of the concrete’ [2] p. 433. This parallels the division that I have advocated for in separating out distinctions related to happening and distinctions related to separability (discussed below). An important point made by Whitehead is that ‘When we divide the satisfaction coordinately, we do not find feelings which *are* separate, but feelings which *might* be separate. In the same way, the divisions of the region are not divisions which *are*; they are divisions which *might be*’ [2] p. 435, which shows that the divisions that he speaks of lie more within the extensive continuum of potentiae rather than the concrete reality of actual occasions.

Whitehead introduced the concept of ‘extensive connection’, although he did not explicitly define it, to the best of my knowledge. He wrote,

The more general type of properties expresses the mere fact of ‘extensive connection’, of ‘whole and part’, of various types of ‘geometrical elements’ derivable by ‘extensive abstraction’; but excluding the introduction of more special properties by which straight lines are definable and measurabilty thereby introduced.

In these general properties of extensive connection, we discern the defining characteristics of a vast nexus extending far beyond our immediate cosmic epoch.

([2] pp. 147–148)

In this final sentence, we see an allusion to a network (nexus) or graphical structure to the extensive connection. Later, in [2], Whitehead more explicitly speaks of connection in relational terms and provides two points of relevance to what I shall show below, namely that these relations are incomplete and intransitive [2] p. 451.

I do not discuss these two models further except to point out that they are precursors of the process spacetime to be described in the following sections. From here on, I wish to focus on the idea of a space or spacetime that is *generated* by process within the specific framework of the process algebra and examine some of its implications for a formal structure of space.

There are many continuous forms of physical spacetime in physics, such as Minkowski space, Robb’s optical order [43], Borcher and Sen’s causal manifolds [59], the Alexandrov topology [18], and general relativity [62], but, since causal tapestries are discrete, the spacetimes that they represent are also discrete. There has been a lengthy debate about discreteness versus continuity [65]. The idea that spacetime could be discrete generally runs afoul of continuous symmetries such as Lorenz invariance. The idea that physical spacetime could be discrete, even finite, gained traction through research into quantum gravity, with concepts such as causal dynamical triangulations [66] and causal sets [67]. Sorkin’s development of causal sets was further inspiration for the approach taken in the process algebra, although the idea of process is not part of his conception. There has been much debate as to whether these causal sets represent the causal generation of spacetime or merely represent certain causal connections in a block-type spacetime. Recent work has focused on the possibility that causal sets actually represent the causation of spacetime [16,68]. However, in these writings, there is no explicit reference to ideas of processes as being the responsible agents.

The idea of a generated spacetime seems contradictory, since time is included within spacetime, but remember that spacetime represents a history, not an ontology, and we only ever know history up to a point. We are forever adding to history. History grows, but this does not mean that reality grows in the same manner. One can create a myriad of structures with a box of Lego pieces, but the number of pieces remains the same—only the structures change. To create new structures, one must decompose the old ones; new structures do not mean additional pieces. This is equally true for the events that make up our reality.

Previously, I argued that the function of a temporal distinction for process is to mark a change, a happening. Could space also be a distinction, and, if so, what could it mark? I shall argue for a conception of spatial distinction based on the assumption that its primary function for process is to enable counting or to mark separability. This idea is not new and has been explored many times in the philosophical literature [64]. Such a spatial distinction could represent physical or geometric space or it could be a more general space, provided that it facilitates counting or separability. Regardless, just like temporal distinctions, spatial distinctions are relations between entities, not entities in their own right. Note that I do not claim that process has anything to do with numbers and counting in general, but that spatial distinctions enable processes to count their actual occasions.

My focus is on actual occasions because it is not clear, in general, that processes themselves can be associated with spatial locations. For example, mind is a process, supervening upon the activity of the central nervous system, but, functionally, mind cannot be associated with a simple point or even a simple region (neurons alone, neurons plus glia, neurons plus glia plus gut microbiome plus neuroimmune hormones, etc.). There are aspects of mind that are associated with actions of the body, with features of the environment, and with the collective activity of a local community of others. Where, exactly, should one localize it to?

Consider a primitive process that generates just a single actual occasion. Would a concept of space have any meaning? How could we tell? Suppose that, in the next generation, this actual occasion is simply replaced in its entirety. All we could know is that a change took place (and perhaps we might not even know this if we could not record a history). All that we might have is merely a temporal distinction. Suppose that the process creates another actual occasion. We might state that the first actual occasion was created ‘there’ in relation to the original actual occasion, but how can we state that the next actual occasion is created at a different ‘there’ relative to either of the preceding actual occasions if only information from the immediately prior actual occasion is propagated forward? Here, we have just a single temporal distinction. However, if information is propagated forward across two generations, then it becomes possible to distinguish two temporal distinctions and to assert a spatial distinction that marks a difference between a single temporal distinction expressed twice (in the case that there is no change in position) and two distinct temporal distinctions where there is a change in position. The spatial distinction marks the presence of two, rather than one.

If a process creates multiple actual occasions in a single generation, then spatial distinctions enable us to assert that there are many, and not merely one, actual occasions present within the generation. This makes sense for entities that can be physically localized, but what of waves, for example, that have no physical localization? Within the process algebra framework, it is interpolation that provides the clue for spatial distinctions. Consider two waves of different frequencies, $A\cos(\omega_{1}x)$ and $B\cos(\omega_{2}x)$. Classically, these can be distinguished by frequency—one type of spatial distinction. However, they may also be distinguished by physical spatial distinctions. If the actual occasions of the first wave are embedded in some causal manifold at $n_{i}^{1}$ and those of the second wave at $n_{i}^{2}$, with these two collections being disjoint, then the two waves are formed by interpolation [3,46] as

(11) $$A\cos\left(\omega_{1}x\right)=\sum_{i}A\cos\left(\omega_{1}n_{i}^{1}\right)g_{i}^{1}\left(x\right)$$

(12) $$B\cos\left(\omega_{2}x\right)=\sum_{i}B\cos\left(\omega_{2}n_{i}^{2}\right)g_{i}^{2}\left(x\right)$$

and their superposition is given by

(13) $$A\cos\left(\omega_{1}x\right)+B\cos\left(\omega_{2}x\right)=\sum_{i}A\cos\left(\omega_{1}n_{i}^{1}\right)g_{i}^{1}\left(x\right)+\sum_{i}B\cos\left(\omega_{2}n_{i}^{2}\right)g_{i}^{2}\left(x\right)$$

where $g_{i}^{j}\left(x\right)$ are the respective interpolation functions.

The spatial distinctions reflected in the embedding sets enable us to distinguish between the two component waves.

## 6. Thereness

Imagine a process having just completed the creation of a generation of actual occasions. To create the next generation, one must first choose an actual occasion within the current generation whose information one wishes to propagate and then initiate the creation of a nascent actual occasion, situating it relationally to the other prior actual occasions. One then propagates information from the prior actual occasion to this nascent actual occasion by selecting an appropriate action from some set of possible actions. There is no preexisting map that can be used to situate this nascent actual occasion. It is one’s action that does so. The action creates a happening and, in so doing, potentially a spatial distinction. The set of actions represents potentialities; the choice and implementation of an action mark the actualization of a potentiality.

In general, many types of action are possible. The first is the direct replacement of a prior actual occasion by a nascent actual occasion, essentially creating an actual occasion ‘here’ with respect to the prior actual occasion. The second may be thought of as situating a nascent actual occasion ‘there’ relative to some prior actual occasion and propagating information there. Repeating the same action with the nascent actual occasion now as prior will propagate information along the same ‘there’, but this is now two generations removed from the initial actual occasion. Continuing in this manner, one may construct a set of actual occasions that are all ‘there’ relative to the initial actual occasion. I call this a *thereness set*. Different initial actual occasions will have different thereness sets generated from the same action.

Here, I abuse standard set notation and consider sequences to be sets whose elements are ordered and where repetition is allowed—for example, the set {x,x,x,x.x,…} or the set $\left\{x_{1},x_{2},x_{3},\ldots \right\}$. I call these q-sets. When order is important, usually, one position will be specified to determine the order; here, this is given by ‘.’—for example, $\left\{x_{1},x_{2},x_{3}.x_{4},x_{5},\ldots \right\}$. q-sets are not essential but do simplify the notation. I also need to slightly weaken the notion of an equivalence class to accommodate thereness. This is because an initial actual occasion lies within every thereness set. In other words, an initial actual occasion is ‘here’ if it is ‘there’ for all of its possible theres.

In order to define a thereness system, we first need the idea of a pointed equivalence relation.

**Definition** **1** (Pointed Equivalence Relation)**.** *Let X be a set and x∈x. A pointed equivalence relation on X with point x is a collection of subsets $\left\{X_{i}\right\}$ of X such that*

*1.*  *$\cup_{i}X_{i}=X$;*
*2.*  *$x\in X_{i}$ for all i;*
*3.*  *The collection $\left\{X_{i}\setminus \left\{x\right\}\right\}$ is pairwise disjoint.*

The collection $\left\{X_{i}\setminus \left\{x\right\},\left\{x\right\}\right\}$ is thus an equivalence relation on *X*.

The above applies to the entire set, but, later, we will need to consider such relations defined only on subsets of the space—hence, we have the following definition.

**Definition** **2** (*x*-based equivalence relation)**.** *Let X be a set and let $X_{x}$ denote a subset of X called the accessible set from x, and let $C_{x}$ be a pointed equivalence relation on $X_{x}$. The collection $\left(C_{x}\setminus \left\{x\right\}\right)\cup \left(X\setminus X_{i}\right)\cup \left\{x\right\}$ is an equivalence relation on X called an x-based equivalence relation on X. A representative of $C_{x}$ is denoted $T_{x}^{i}$, so we denote the equivalence relation by $\left(X_{x},C_{x}\right)$ (or inaccurately but simply by $\left(X_{x},T_{x}^{i}\right)$).*

**Definition** **3** (Thereness Systems)**.** *Let X be a set and, for each x∈X, let $\left(X_{x},T_{x}^{i}\right)$ denote an x-based equivalence relation on X. For any member of a pointed equivalence relation, $T_{x}^{i}$, set $T_{x}^{i}\setminus \left\{x\right\}=T_{x}^{*i}$. This collection forms a thereness system on X if the following conditions are met:*

*1.*  *For all x,y∈X, if $y\in X_{x}$, then $X_{y}\subseteq X_{x}$;*
*2.*  *For all x∈X, $x\in X_{x}$ and for all i, $x\in T_{x}^{i}$;*
*3.*  *For all x,y∈X, if $x\in X_{y}$ and $y\in X_{x}$, then x=y;*
*4.*  *If $y\in T_{x}^{i}$, then there is at most one j such that $T_{y}^{*j}\cap T_{x}^{i}\neq \emptyset$, in which case $T_{y}^{j}\subseteq T_{x}^{i}$;*
*5.*  *If y,z are such that $T_{y}^{j}=T_{z}^{k}$, then y=z and j=k;*
*6.*  *If $y,z\in T_{x}^{i}$, then there exist j,k such that either $T_{y}^{j}\subseteq T_{z}^{k}$ or $T_{y}^{j}\subseteq T_{z}^{k}$ and $T_{y}^{j},T_{z}^{k}\subseteq T_{x}^{i}$;*
*7.*  *If x,y,w,z are distinct elements such that $\left\{w,z\right\}\subset T_{x}^{i}\cap T_{y}^{j}$, then either $T_{x}^{i}\subseteq T_{y}^{j}$, or $T_{y}^{j}\subseteq T_{x}^{i}$.*

The rationale for the above definition is as follows, with each item referring to its corresponding item in the above definition.

1. This is a transitivity condition that states that, if *y* is accessible from *x* and *z* is accessible from *y*, then *z* is accessible from *x*.
2. It is possible for there to be no elements at a particular there from an element *y*, but I consider *y* itself to always be there for every possible there, which is what makes it here.
3. If *y* is accessible from *x* and *x* is accessible from *y*, then they must be the same element. This is because any element will necessarily be there only at some future time. The only element for which this is not true is itself. This is in keeping with the temporal irreversibility of process.
4. This is also a more refined transitivity condition that ensures that, if *y* is accessible there from *x* and *z* is accessible there from *y*, then *z* is accessible there from *x*.
5. This states that there cannot be two different theres from *y* that match the there from *x*; the theres must align consistently. Another way to state this would be if $y\in T_{x}^{i}$ and $T_{y}^{*j}\cap T_{x}^{i}\neq \emptyset$, $T_{y}^{*k}\cap T_{x}^{i}\neq \emptyset$, then j=k.
6. This condition ensures that each thereness is distinct from another, so, if two thereness classes from the same element should intersect, then they are the same. In other words, two thereness classes can have only one point in common, which is the point of the equivalence relation.
7. This condition ensures that the elements of a thereness class are totally ordered. This will be apparent in the next section when thereness classes are generated by the actions of process, but it also is in keeping with the sense that the concept of there has a linearity associated with it.
8. This condition asserts that, if the theres from two distinct elements overlap in more than a single point, thus corresponding to an interval of thereness, then the two theres must be aligned. This merely expresses the usual requirement that two lines should intersect only at single points, if at all.

**Theorem** **1.** *Let the set X be endowed with a thereness relation A. For any x∈X and index i∈I, $T_{x}^{i}$ is totally ordered, and x is minimal in the order.*

**Proof.**  Let *X* be a q-set and pick any x∈X, i∈I. Define an order on $T_{x}^{i}$ by y<z if there exist indices j,k such that $T_{z}^{j}\subset T_{y}^{k}$. Equally, y<z if $z\in T_{y}^{j}$. Condition 4 above ensures that, for any two elements in $T_{x}^{i}$, either y<z or z<y. If y<z and z<y, then $T_{y}^{j}\subset T_{z}^{k}$ and $T_{y}^{j}\subset T_{z}^{k}$. However, this implies that $T_{y}^{j}=T_{z}^{k}$. By the above, y=z. Clearly, *x* is minimal, since, for any *y*, $T_{y}^{j}\subset T_{x}^{i}$. □

Suppose that $y\in T_{x}^{i}$. Being a pointed equivalence relation, there must exist some *j* such that $x\in T_{y}^{j}$. Suppose that $T_{y}^{j}\setminus T_{x}^{i}\neq \emptyset$. There must exist $z\in T_{y}^{j}\setminus T_{x}^{i}$ and *k* such that $z\in T_{x}^{k}$. Clearly, $T_{x}^{k}\subset T_{y}^{j}$. $T_{y}^{j}$ is thus the reverse direction to $T_{x}^{i}$ from *y*, and so $T_{x}^{k}$ is thus the reverse direction to $T_{x}^{i}$. Thus, to each thereness direction, we can associate a reverse thereness direction. The thereness direction from *y* to *x* is thus the reverse of the thereness direction from *x* to *y*. I denote the reverse thereness direction to $T_{x}^{i}$ by $T_{x}^{-i}$.

Given a single element {x}, the equivalence class is a singleton, $T_{x}^{a_{0}}=\left\{x\right\}$. There is only here, and every there is just here. If there are two elements, x,y, then there are three possibilities. We could have $T_{x}^{a_{0}}=\left\{x\right\},T_{y}^{a_{0}}=\left\{y\right\}$, or we could have $T_{x}^{a_{0}}=\left\{x\right\},T_{x}^{a_{1}}=\left\{x,y\right\},T_{y}^{a_{0}}=\left\{y\right\}$, or we could have $T_{x}^{a_{0}}=\left\{x\right\},T_{y}^{a_{1}}=\left\{y,x\right\},T_{y}^{a_{0}}=\left\{y\right\}$. In the case of three elements, we might have $T_{x}^{a_{0}}=\left\{x\right\},T_{x}^{a_{1}}=\left\{x,y,z\right\},T_{y}^{a_{0}}=\left\{y\right\},T_{y}^{a_{2}}=\left\{y,z\right\},T_{z}^{a_{0}}=\left\{z\right\}$. Obviously, the situation becomes rapidly more complicated as the number of elements increases.

Note that this ‘thereness’ is a complex mixture of time and space. It involves both a transition in time—a shift from one generation to another, and therefore a duration—and a shift in space, or a generation of a locale in space. In order to separate the spatial aspect from the temporal, we must go one step further.

## 7. Process as Generator of Space

Previously, I suggested that processes were generators of space. In particular, the actions attributed to processes in the preceding section give rise to a system of thereness. This will be demonstrated in this section. Let us denote the set of actions used by a process to initiate and to propagate information to nascent actual occasions by $A=\{a_{0},a_{1},a_{2},a_{3},\ldots \}$, where, apart from $a_{0}$, the index has no meaning apart from being an index.

The action $a_{0}$ is the unique action described in the previous section that propagates information to an actual occasion and directly replaces the prior actual occasion that originated this action. It is the action that propagates information ‘here’ rather than ‘there’. It can be thought of as the stationary action, or the here action. Note that, for all actions $a_{i}$, we have $a_{0}a=aa_{0}$, because, logically speaking, staying still for one generation and then moving must be the same as moving for one generation and then staying still. This will be important when considering the algebra of spatial distinctions created by a process.

Note that each action will situate the nascent actual occasion in the succeeding generation from that of the actual occasion initiating the action. In any equation involving actions, it is important that the generation numbers on either side of the equation match.

By definition, an entity is inertial if it is either at rest or in uniform motion, i.e., not under the influence of an external force. A process can be expected to be inertial if it is not in interaction with an external process. It is reasonable in such a case to assume that the set of actions available to the process does not change over time, which would not be the case in the presence of interactions with an external process. This suggests the following.

**Definition** **4** (Inertial Process)**.** *A process is inertial if there exists a single set A of actions $\left\{a_{0},a_{1},a_{2},\ldots \right\}$ that applies to all generated actual occasions, irrespective of generation or spatial distinctions. More precisely, the algebraic relations among the actions in A should be independent of the actual occasion upon which they act.*

A process, therefore, is non-inertial if its action set is linked to individual actual occasions, and this only occurs when the process is interacting with other, external processes. An inertial process generally manifests temporal translational and spatial translational symmetry. Temporal translational symmetry is expected by Noether’s theorem, since inertial systems are expected to exhibit conservation of energy, and it can be expressed in the assertion that $a_{0}^{n}a=aa_{0}^{n}$ for all n,a. Spatial translational symmetry may add a slightly different factor, separate from the spatial distinctions among actual occasions. It may also include the idea that, if one can propagate information from some spatial position *r* to a position *s*, then one should also be able to propagate information from position *s* to position *r*. Thus, one can return to one’s previous position, although one cannot return to one’s original generation. This weak form of temporal symmetry was described by Hardy [69] and is expressed here by asserting that, for any action $a_{i}$, there should exist an action $a_{-i}$ such that $xa_{i}a_{-i}=xa_{-i}a_{i}=xa_{0}^{2}$. The exponent 2 is required because this requires two generations to unfold. Of course, there may be processes in which such reverse actions are not present, but, for this paper, we shall assume that they do indeed exist for all actions. Note that temporal translational symmetry does not imply temporal reversal symmetry. Time reversal is not allowed in process theory. Information can only flow between immediate prior and nascent generations. It cannot flow from generation *n* to generation n+k for k>1 because generation n+k does not exist when generation *n* exists (it has not yet been generated), and generation *n* does not exist when generation n+k exists because it ceases to exist when the concrescence of generation n+1 is complete. Thus, time reversal is impossible. Closed causal loops and other forms of time reversal [42] are not possible in a process world.

This should be distinguished from the function $a^{-1}$, which is not an action and applies only to histories of processes. It is the function defined by $xa^{-1}=y$ such that ya=x. It points to the prior actual occasion that propagated information ‘there’ to the current actual occasion. Clearly, this cannot be a process action since information is only ever propagated forward in generation number.

However, there is subtlety here. Strictly speaking, the actions *a* referred to above can be thought of as *actualization* actions; they mark the initiation of the concrescence of a nascent actual occasion and establish its spatial distinctions with the other prior actual occasions (and whatever nascent actual occasions there are undergoing concrescence). The propagation of information from prior actual occasions to nascent actual occasions is carried out by *p* actions (for *propagation*). It is possible for actions to act in a backwards manner from nascent actual occasions to prior actual occasions to influence the choice of *p* action, resembling Bergson’s concept of *interpenetration* [70], in which there is an interplay between incoming information and emerging interpretations of this information (the subjective experience of music is often given as an example). These are carried out by *c* (for *concrescence*) actions. These backward actions do not affect the information content of prior actual occasions and, strictly speaking, occur contemporaneously with *p* actions and not retroactively, and so there is no backward propagation of information in time. These *c* actions might select information for inclusion (Whitehead’s positive prehensions) or reject certain information (negative prehensions). Formally, while one can concatenate two *p* actions, one cannot concatenate two *c* actions, so that $c_{i}c_{j}=\emptyset$ for all i,j. This subtlety is not important for the construction of the process spacetime discussed here, which relies solely on *a* actions, but it is a consideration when constructing a model of concrescence (in which case they are merely heuristic devices). This interplay between past and future events is a feature of some interpretations in quantum mechanics, such as Cramer’s transactional interpretation [71] or Aharonov’s two-time physics [72].

Actions are combined through their sequential application. Thus, finite sequences $a_{1}a_{2}\cdots a_{n}$ of actions are also actions, but they now act across multiple generations—in the above case, *n* generations—rather than single generations. Thus, these are not actions that can be undertaken by the process as it creates a nascent generation, but they can be applied to any history of the process and thus any causal tapestry of process history.

The collection of all possible actions thus forms formally the free semigroup $A=\{a_{i_{1}}^{n_{1}}\cdots a_{i_{k}}^{n_{k}}|a_{i_{j}}\in A\}$ with base set A, subject to the constraints $a_{i}a_{0}=a_{0}a_{i}=a_{0}^{2}$ for all $a_{i}\in A$. We may turn A into a free monoid by adding an additional, formal ‘action’, 1, which acts as an identity when applied to any action or actual occasion. It is equivalent to the action ‘do nothing’, which, while not something that a process actually does, nevertheless turns out to be useful formally.

It may be the case that not all actions can be utilized upon a given actual occasion. For example, if the action *a* cannot be applied to actual occasion *x*, we set xa=∅. Thus, we can set $X_{x}=xA$ for all *x*.

There may be additional algebraic relations among the actions. For example, it may be the case, given an initial actual occasion *x* and actions a,b,c,d, that xab=xcd, meaning that applying the actions results in the same actual occasion being created. In this case, we can identify the actions ab and cd and set ab=cd as an additional algebraic relation in A.

In order to see how process actions generate spatial distinctions, I give the following definition.

**Theorem** **2** (Action-Based Thereness System)**.** *Let Π be a process and A be a monoid of action operators $A=\{a_{0},a_{1},a_{2}\ldots \}$. A thereness class $T_{x}^{a_{i}}=\left\{x,xa_{i},xa_{i}a_{i},xa_{i}a_{i}a_{i},\ldots \right\}=\left\{x,xa_{i},xa_{i}^{2},xa_{i}^{3},\ldots \right\}$. A is called a generator of a thereness system and the set of all thereness classes forms a thereness system if the following conditions hold:*

*1.*  *$X_{x}=xA$ for all actual occasions x;*
*2.*  *For all actual occasions x, actions $a_{i}$, and generations n, if x lies in generation n, then $xa_{i}$ lies in generation n+1;*
*3.*  *There is a unique operator, $a_{0}$, such that $a_{0}a_{i}=a_{i}a_{0}$;*
*4.*  *For all actual occasions x,y and any $a_{i}$, $xa_{i}=ya_{i}$ implies that x=y;*
*5.*  *For all informons x and actions $a_{i},a_{j}$ and any k>0, $xa_{i}^{k}=xa_{j}^{k}$ implies that i=j;*
*6.*  *For all actual occasions x and actions $a_{i}$, there exists an action $a_{-i}$ (called the reverse of $a_{i}$) such that $xa_{i}a_{-i}=xa_{-i}a_{i}=a_{0}^{2}$.*

**Proof.**  Let Π be a process, *X* its set of actual occasions, and $A=\{a_{0},a_{1},a_{2}\ldots \}$ its monoid of actions. Let $T_{x}^{a}=\left\{x,xa_{i},xa_{i}a_{i},xa_{i}a_{i}a_{i},\ldots \right\}$ and set $X_{x}=xA$.

1. If $y\in X_{x}$, then y=xb for some b∈A. If $z\in X_{y}$, then z=yc for some c∈A. Then, $z=yc=xbc\in X_{x}$.
2. Let x,y∈X. Assume that $x\in X_{y}$ and $y\in X_{x}$. Then, for some actions, a,b and i,j, $x=ya^{i}$, $y=xb^{j}$; then, $x=xb^{j}a^{i}$, so that i=j=0.
3. Let $y\in T_{x}^{i}$; then, for some action *a* and *i*, $y=xa^{i}$, so the set $T_{y}^{j}=\left\{y,ya^{1},ya^{2},ya^{3},\ldots \right\}$ will satisfy $T_{y}^{j}\subseteq T_{x}^{i}$, and it is clearly unique.
4. If y,z satisfy $T_{y}^{i}=T_{z}^{j}$, then, for some actions a,b and n,m, $y=za^{n}$ and $z=yb^{m}$. Then, $y=yb^{m}a^{n}$, which implies that n,m=0 and hence y=z.
5. Let $y,z\in T_{x}^{i}$. Then, there exist n,m and action *a* such that $y=xa^{n}$ and $z=xa^{m}$. Without loss of generality, assume that m<n. Then, $y=xa^{m}a^{n-m}=za^{n-m}$, so $T_{y}^{i}\subseteq T_{z}^{j}$ for some i,j.
6. Suppose that there are distinct elements x,y,w,z such that $\left\{w,z\right\}\in T_{x}^{i}\cap T_{y}^{j}$. Then, for some n,m,r,s and actions a,b, we have $w=xa_{i}^{n}=ya_{j}^{m}$ and $z=xa_{i}^{r}=ya_{j}^{s}$. If g() denotes the generation number of an actual occasion, then, first of all, note that the generations must match. That is, g(x)+n=g(y)+m and g(x)+r=g(y)+s. Subtracting, we find n−r=m−s. Without loss of generality, assume that g(w)>g(z), so that n−r>0. Then, $w=xa_{i}^{r}a_{i}^{n-r}=ya_{j}^{s}a_{j}^{m-s}$, so that $w=za_{i}^{k}=za_{j}^{k}$, where n−r=m−s=k. Then, from condition 4 above, i=j. This implies that $T_{x}^{i}\cap T_{y}^{i}\neq \emptyset$. In particular, this implies that, for some $k,k^{\prime }$, $xa_{i}^{k}=ya_{i}^{k^{\prime }}$. Again, without loss of generality, assume that g(y)>g(x). Clearly, $g\left(x\right)+k=g\left(y\right)+k^{\prime }$. So $xa_{i}^{\prime k}a_{i}^{k-k^{\prime }}=xa_{i}^{k-k^{\prime }}a_{i}^{k^{\prime }}=ya_{i}^{k^{\prime }}$. Applying condition 3 repeatedly, we obtain that $xa_{i}^{k-k^{\prime }}=y$, so that $T_{y}^{i}\subset T_{x}^{i}$.

□

To justify the suggestion that this final condition provides a form of spatiotemporal symmetry, assume that an interpretation of the process and its actual occasions is available that maps to some *n*-dimensional real manifold. Furthermore, assume that the effect of an action can be represented by a vector action. If the actual occasion *x* is interpreted as having position x and the action *a* is interpreted as propagating to a position offset by a vector w, then xa=x+w. Let the offset for *a* be w, for $a_{-i}$ be $w^{\prime }$, and for $a_{0}$ be v. Then, given an actual occasion x, we have $xa_{i}a_{-i}=x+w+w^{\prime }=x+2v=xa_{o}^{2}$. If we translate to a new reference frame by −v, then we get $x-v+w-v+w^{\prime }-v=x-v+2v-2v$, so that $\left(w-v\right)+\left(w^{\prime }-v\right)=0$. This implies that $\left(w-v\right)=-\left(w^{\prime }-v\right)$, so $a_{-i}$ does act like a spatial inverse to $a_{i}$.

Thus, $a_{0}$ does act like a zero vector. However, it is very important to note that it acts as a zero vector only with respect to a single process. There is no reason, a priori, that any directionality imparted to $a_{0}$ as it acts within a process Π should be the same directionality when it acts within a second process $\Pi^{\prime }$. These actions operate locally, resulting in only a local spacetime structure.

**Theorem** **3** (Parallelism)**.** *Let Π be a process such that its set of actual occasions X, together with its action monoid set, $A=\{a_{0},a_{1},a_{2}\ldots \}$, forms a thereness system. Then, for any x,y∈X, x≠y, and a∈A, if $T_{x}^{a}\cap T_{y}^{a}\neq \emptyset$, then either $T_{x}^{a}\subseteq T_{y}^{a}$ or $T_{y}^{a}\subseteq T_{x}^{a}$.*

**Proof.**  Suppose that there exist x,y∈X, x≠y, and a∈A such that $T_{x}^{a}\cap T_{y}^{a}\neq \emptyset$. Then, for some n,m, we have $z=xa^{n}=ya^{m}$. Without loss of generality, assume that m<n. Then, $za^{n-m}a^{m}=ya^{m}$. Since *a* is 1-1, so must be $a^{m}$; hence, $y=xa^{n-m}$. □

We can now describe the structure of spacetime as generated by a process. Recall the temporal structure described above. It takes the form

(14) $$\cdots ⊕G\left(A_{i},A_{i+1}\right)⊕G\left(A_{i+1},A_{i+2}\right)⊕G\left(A_{i+2},A_{i+3}\right)⊕\cdots$$

where each $A_{i}$ is an anti-chain of actual occasions and each $G(A_{k},A_{k+1})$ is a directed graph with initial vertices in $A_{i}$ and terminal vertices in $A_{i+1}$, and ⊕ is a graph sum.

Each directed edge represents an instance of information propagation from a prior to a nascent actual occasion. Let us label each such edge by the specific action undertaken in the propagation of said information. That is, if *n* is a prior actual occasion and *m* a nascent or immediately subsequent actual occasion, and information was propagated from *n* to *m* via the action *a*, then we label the edge n→m as $n\overset{a}{\to }m$. We do this for every directed arrow in the above graph.

Consider now a labeled mixed multigraph in which the directed edges occur singly, while undirected edges may occur multiply. That is, given a set of vertices *V*, we have a set of labeled directed edges *T* on *V* together with a set *S* of labeled undirected edges on *V*. One can guess that *T* refers to timelike and *S* to spacelike. If *V* is a set of actual occasions generated by a process, *T* is the set of labeled directed edges of the form $x\overset{a}{\to }y$ if xa=y. The set *S* is constructed as follows. Given two actual occasions $m_{1},m_{2}$ appearing in the same generation, we add an edge $m_{1}\overset{\left(a_{1},a_{2}\right)}{-}m_{2}$ (*Consanguinity edge*) if there is a prior actual occasion *n* and actions $a_{1},a_{2}$ such that there already exist labeled directed edges $n\overset{a_{1}}{\to }m_{1}$ and $n\overset{a_{2}}{\to }m_{2}$ in the set *T*. There may be many such prior actual occasions and actions, which is why *S* becomes a multigraph. I denote the resulting labeled multigraph as

(15) $$\cdots {\hat{A}}_{i}⊕G\left({\hat{A}}_{i},{\hat{A}}_{i+1}\right)⊕{\hat{A}}_{i+1}⊕G\left({\hat{A}}_{i+1},{\hat{A}}_{i+2}\right)⊕{\hat{A}}_{i+2}⊕G\left({\hat{A}}_{i+2},{\hat{A}}_{i+3}\right)⊕{\hat{A}}_{i+3}⊕\cdots$$

This graph is the called the graph of spatial and temporal distinctions, or the process spacetime history, of the process. In what follows, I may abuse the notation slightly and refer to the graph as a process spacetime or process spacetime graph, but it should always be understood as representing the history of a process. A process always generates a compound present and never a block universe structure.

**Figure 2:**
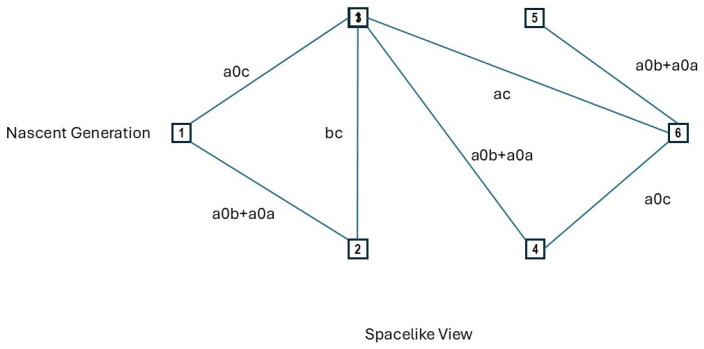
Spacelike view. This figure depicts the nascent generation of Figure 1, showing its spacelike relations. The letters refert to process actions. For simplicity, the action label (a,b) is depicted as ab, and, if there are multiple edges between vertices with labels, say, (a,b),(c,d), this is depicted as ab+cd. Two actual occasions share an edge if and only if they have a common prior actual occasion.

The undirected edges of type $m_{1}\overset{\left(a_{1},a_{2}\right)}{-}m_{2}$ noted above do not represent actions on the part of the process because they do not involve any transfer of information between the related actual occasions. Nevertheless, they convey spatial distinctions between the related actual occasions, inherited from the identified actions, and, because they arise from common causes, they also potentially convey information about correlations in the information conveyed by these two actual occasions. Figure 1 and Figure 2 depict a simple example of a timelike and spacelike view (subgraphs) of a single compound present formed of a prior and a nascent generation. The complete spacetime graph is formed by taking the graph union of these two subgraphs (views).

Note that some actual occasions do not have an edge linking them as they share no common cause. Any ‘distance’ attributed to them is inherited from the causal manifold that is used to interpret them—it is not necessarily intrinsic. This is a departure from the usual understanding of ‘space’ because, from a process perspective, spacelike links arise from correlations among associated information. The actual occasions are spatially distinguished functionally—not via some absolute geometry. It is a spacetime history based upon both ontology—that is, how the actual occasions are spatially distinguished as a result of their creation by some process—and also epistemology, since there must be an actual transfer of information to form a link and not some declaration from omniscience that cannot be verified directly.

Each prior actual occasion will generate a complete subgraph in the nascent generation graph. However, a given prior actual occasion need not contribute to every actual occasion in the nascent generation. Thus, the labeled multigraph for a generation will consist of a collection of complete subgraphs with incomplete connectivity between them. This may be important in understanding entanglement and contextuality [73], where issues of intransitivity and incompleteness play a role.

The undirected edges correspond to spacelike links between events in the case of a physical spacetime, but, more generally, they refer to possible correlations between the connected events. Unlike in a physical spacetime, in which any two events have a distance assigned between them, not all events in a process spacetime will have edges linking them due to the absence of any common cause in their generation. These links are related to information flows created by processes. They are absent from traditional physical spacetimes but may play important roles in some perplexing quantum behavior, such as entanglement. Feynman pointed out that, for the most part, physics does not consider any role for history in the dynamics of its entities. He felt that this might be a serious shortcoming [74]. In a strict deterministic dynamics, any single state possesses all of the information needed to determine both its future evolution and its prior history. This is not the case for process. It is impossible to determine these correlational links between the spacelike separated actual occasions within a generation without knowledge of the history leading up to the generation of these actual occasions. The existence of these correlations is lacking in our traditional spacetime representations, but it is made explicit in process spacetimes, suggesting that the hidden nature of these correlations is an artifact of our means of representation and not a matter of ontology.

## 8. Position

Process actions always involve both space and time—at the very least, a change in generation and sometimes a change in spatial distinctions. Temporal distinctions, at a minimum, may be expressed by the generation number. Spatial distinctions are implicit but can be made explicit via the idea of position. Recall that there is a privileged action, $a_{0}$, which is the action that directly replaces a prior actual occasion with a nascent actual occasion. It has the property that it commutes with every other action, $aa_{0}=a_{0}a$, and, for every action $a_{i}$, we have an action $a_{-i}$ such that $a_{i}a_{-i}=a_{-i}a_{i}=a_{0}^{2}$.

Given any action *a*, we can define $xa^{-k}=y$ such that $ya^{k}=x$. If there is no such element, we set $xa^{-k}=\emptyset$. Note that $a_{0}a_{0}^{-1}=a_{0}^{-1}a_{0}=1$, since concatenating these two actions is effectively the same as doing nothing.

Let *x* be an actual occasion and consider the bi-infinite sequence

$$\ldots ,xa_{0}^{-3},xa_{0}^{-2},xa_{0}^{-1},x1,xa_{0},xa_{0}^{2},xa_{0}^{3},\ldots$$

or, equally, one could consider the q-set $\left\{\ldots ,xa_{0}^{-3},xa_{0}^{-2},xa_{0}^{-1},x1,xa_{0},xa_{0}^{2},xa_{0}^{3},\ldots \right\}$.

We let either the sequence or the q-set represent the *position* of *x* and denote it as $P_{x}$.

Note that

$$P_{x}a_{0}=\ldots ,xa_{0}^{-3}a_{0},xa_{0}^{-2}a_{0},xa_{0}^{-1}a_{0},xa_{0},xa_{0}a_{0},xa_{0}^{2}a_{0},xa_{0}^{3}a_{0}=$$

$$\ldots ,xa_{0}^{-2},xa_{0}^{-1},x1,xa_{0},xa_{0}^{2},xa_{0}^{3},\ldots =P_{x},$$

(noting that $a_{0}^{-k}a_{0}=a_{0}^{-k+1}$), which is what one would expect for the concept of a position.

Moreover, note that, for any actual occasion *x* and action *a*,

$$P_{x}a=\ldots ,xa_{0}^{-3}a,xa_{0}^{-2}a,xa_{0}^{-1}a,xa,xa_{0}a,xa_{0}^{2}a,xa_{0}^{3}a,\ldots =$$

$$\ldots ,xaa_{0}^{-3},xaa_{0}^{-2},xaa_{0}^{-1},xa,xaa_{0},xaa_{0}^{2},xaa_{0}^{3},\ldots =P_{xa},$$

so an action *a* propagates information from an actual occasion at position $P_{x}$ to an actual occasion at position $P_{xa}$.

We say that two actual occasions x,y occupy the same position if $y\in P_{x}$ (equally, $x\in P_{y}$). This makes sense since, clearly, one reaches the other by repeated applications of the stationary action $a_{0}$, even if this is not reversible. One can see this by simply moving the position of the signifier ‘1’ to the *y* and relabeling the remaining terms. That is,

$$\ldots ,xa_{0}^{-3},xa_{0}^{-2},xa_{0}^{-1},x1,xa_{0},xa_{0}^{2},xa_{0}^{3},\ldots ,xa_{0}^{k-1},y=xa_{0}^{k},xa_{0}^{k+1},xa_{0}^{k+2},\ldots$$

becomes

$$\ldots ,ya_{0}^{-2-k},ya_{0}^{-1-k},ya_{0}^{-k},ya_{0}^{1-k},ya_{0}^{2-k},ya_{0}^{3-k},\ldots ,ya_{0}^{-1},y1,ya_{0}^{1},ya_{0}^{2},\ldots$$

Let $a_{i}$ be an action, $a_{-i}$ its reverse, and *x* any actual occasion. Then,

$$P_{x}a_{i}a_{-i}=P_{x}a_{0}^{2}=\left(P_{x}a_{0}\right)a_{0}=P_{x}a_{0}=P_{x}$$

so that the action of $a_{-i}$ on a position is actually the reverse of the action of ai.

We thus have a primitive time, as reflected in the generation number, and a primitive space, as reflected in the position. Note that we have not yet introduced any metric structure (only the generation number is fixed; the durations are as yet unspecified), nor have we ascribed any additional algebraic relations to these positions, such as a vector structure. Metricity, and the algebraic structure of the positions, will depend upon the nature of the processes being modeled and is left for a subsequent paper.

## 9. Commutative Space

In the process setting, the concept of position is emergent; it is not a fundamental concept. Spatial distinctions are generated by process actions. As noted previously, under concatenation, the set of all actions becomes a free monoid, with 1 as the identity. A more complicated structure arises from constraints, such as having a distinguished action $a_{0}$ subject to the constraint $aa_{0}=a_{0}a=a_{0}^{2}$ for all actions *a*. Additional constraints, such as ab=cd, as noted previously, may arise when considering specific processes and give the structure its specific geometry. Moreover, the finite existence of most processes implies that, in general, one can expect that concatenations will be of finite length. Different processes will have different action sets and so create different spacetime structures (for example, processes corresponding to different velocities will have different action sets subject to relativistic constraints).

It is difficult to discuss the most general case at length other than to note that every possible concatenation will potentially give rise to a different position once a collection of initial actual occasions has been created. Given an actual occasion *x*, the collection

$$\left\{P_{xa_{i_{1}}^{n_{1}}\cdots a_{i_{k}}^{n_{k}}}|a_{i_{j}}\in A\right\}$$

forms a collection of possible positions.

Here, I would like to comment on what is likely the simplest case of constraints on the free monoid, namely ab=ba for every a,b∈A, which forces it to become an Abelian monoid. Commutativity greatly simplifies the structure of the space.

From now on, we assume that A is a commutative monoid and let *a* be an action.

We can form three different sequences or q-sets:

1. (Hence set) $H\left(a\right)=\left\{1,a,a^{2},a^{3},\ldots \right\}$;
2. (Whence set) $W\left(a\right)=\left\{\ldots ,a^{-3},a^{-2},a^{-1},1\right\}$;
3. (Direction set) $D\left(a\right)=\left\{\ldots ,a^{-3},a^{-2},a^{-1},1,a,a^{2},a^{3},\ldots \right\}$.

Note that a thereness class is formed by applying a hence set to an actual occasion, $T_{x}^{a}=xH\left(a\right)$.

A hence set corresponds to the future set from an actual occasion along a there given by the action *a*. A whence set provides a past set leading to an actual occasion along a there given by the action *a*, while the direction set gives the entire range of past and future actual occasions corresponding to a given there determined by the action *a*.

Consider a hence set as an infinite vector (although, in reality, all three sets will generally be effectively finite since the entries will be ∅ for all but a finite number). Now, take two such sets, H(a),H(b), and consider pointwise multiplication, with a∅=∅a=∅ for all *a*,

(16) $$H\left(a\right)★H\left(b\right)=\left\{1,a,a^{2},a^{3},\ldots \right\}★\left\{1,b,a^{2},b^{3},\ldots \right\}=\left\{1,ab,a^{2}b^{2},a^{3}b^{3},\ldots \right\}=$$

$$\{1,ab,{\left(ab\right)}^{2},{\left(ab\right)}^{3},\ldots \}=H\left(ab\right)$$

Note that a thereness set $T_{x}^{a}=xH\left(a\right)$. Thus, thereness inherits some of the algebraic structure of the actions that generate it.

We can perform the same multiplication with whence and direction sets, so W(a)★W(b)=W(ab) and D(a)★D(b)=D(ab), and it is obvious that, for any actual occasion *x*, $P_{x}=xD\left(a_{0}\right)$. In other words, a position is the direction associated with the stationary action.

**Theorem** **4.**
  *Let Π be a process with a monoid set of actions A. If the monoid operation on A is commutative, then the thereness space has the structure of a discrete lattice and thus is flat.*

**Proof.**  Any element in A will take the form $a_{i_{1}}^{n_{1}}\cdots a_{i_{k}}^{n_{k}}$ for some $a_{i_{j}}\in A$ and $n_{i}$. Since the monoid is commutative, we can rearrange each concatenation so that each $a_{i}\in A$ occurs at most once, raised to some nonzero power. Moreover, we can arrange it so that each action $a_{i}$ is paired with its reverse. Thus, each concatenation now takes the form $a_{i_{1}}^{n_{1}}a_{-i_{1}}^{n_{1}^{\prime }}\cdots a_{i_{k}}^{n_{k}}a_{-i_{k}}^{n_{k}^{\prime }}$. Now, note that we can effectively eliminate any pair $a_{i}^{n_{i}}a_{-i}^{n_{i}^{\prime }}$, replacing it with ${\hat{a_{i}}}^{|n_{i}-n_{i}^{\prime }|}$, where $\hat{a_{i}}=a_{i}$ if $n_{i}>n_{i}^{\prime }$ and $\hat{a_{i}}=a_{-i}$ if $n_{i}^{\prime }>n_{i}$. If $n_{i}=n_{i}^{\prime }$, we can obviously eliminate both $a_{i}$ and $a_{-i}$. If we do so, each concatenation now takes the form ${\hat{a}}_{i_{1}}^{n_{1}}\cdots {\hat{a}}_{i_{k}}^{n_{k}}$, where $\hat{a}$ means either *a* or $a_{-}$, but not both, and every $n_{k}$ is nonzero.

Since $a_{-i}$ is effectively the reverse there to $a_{i}$, we can map the spatial distinctions to a discrete lattice, where each direction is given by some $\hat{a_{i}}$, and the position along this direction is given by the exponent in the positive direction *n* if there is a term $a_{i}^{n}$ in the concatenation and *n* units in the negative direction in there is a term $a_{-i}^{n}$. This suggests that, in the case of a commutative monoid of actions, the resulting spatial distinctions are structured in the form of a discrete lattice. Commutativity thus gives rise to a flat space. □

## 10. Process Action, Interaction, and Contextuality

The previous sections focused on the situation in which a single process was active and observed how its creation of actual occasions resulted in the creation of a structure of spatial and temporal distinctions—a spacetime history. The situation of multiple processes is challenging. Independent processes result in spacetime histories that may also be independent of one another. Each history is local to its process; there need be no synchronization in the timing of the creation of generations, or of individual actual occasions, or in the use of individual actions. Each independent process will propagate its actual occasions inertially, generating its own local directionality. Different processes will generate different such directionalities. One way to determine these relative directionalities would be through processes sending signals to one another, although this would have the potential to destroy their independence. Another way would be to adopt an omniscient vantage point, although, again, this can be adopted by a mathematician but not necessarily any other observer. Another way is to assume that processes do not arise from nothing (the Big Bang aside) and to attempt to trace back the long-term history of these processes to some primordial ancestor that might have set the first prime direction.

The situation is simpler when processes interact, since there are now constraints over the creation of actual occasions set via the process algebra.

In constructing the spacetime histories of both single and interacting processes, two principles must be followed.

1. The Principle of Ontological Separateness or Non-Ambiguity: No two actual occasions of the *same* process may be assigned the same spatial and temporal distinctions.
2. The Principle of Exclusion or Distinguishability: No two processes of the *same* type (same intrinsic characteristics) may assign actual occasions to the same temporal and spatial distinctions.

As an example of the first principle, it is assumed that no photon can generate actual occasions corresponding to two different energy states *at the same location*. As an example of the second principle, no two fermions of the same spin, rest mass, energy, etc., can assign actual occasions *to the same location*. One ambiguity may be possible in that actual occasions from different processes may be assigned the same spacetime location provided that there are additional spatial distinctions that distinguish them. For example, an actual occasion from a boson and one from a fermion might potentially be created at the same location since they may be clearly distinguished from one another.

Thus, several actual occasions may be assigned the same temporal and spatial characteristics so long as there exist additional spatial distinctions that distinguish them from one another. In such a case, we can think of these additional spatial distinctions as providing additional ‘dimensions’, and actual occasions may be ‘stacked’ at a given spacetime locus, similarly to stacking game pieces, one on top of another, in a game of checkers. These additional spatial distinctions ensure that, given the *totality* of temporal and spatial distinctions, only one actual occasion is ever associated with a particular spacetime location. In this way, ontological separability holds in all cases, and information flow among the processes can remain coherent.

Interacting processes add a layer of complexity to the process spacetime graph. In process sums (superpositions), actual occasions are generated sequentially and any relationship between generated actual occasions is via either consanguinity edges or via the property vector that links each actual occasion to its generating process. In process products, however, actual occasions are generated concurrently by the sub-processes involved. To account for this (important for entanglement), we add additional unlabeled, undirected edges between actual occasions $m_{1},m_{2}$ if they are generated concurrently (*Concurrency edges*).

Interacting processes (nexuses) are represented by a process interaction graph $G(\Pi_{i})$, which is an edge-labeled graph whose vertex set consists of the processes $\Pi_{i}$ and whose edge set consists of all pairs of vertices whose processes are engaged in some interaction, while the label is the operator in the process algebra that defines the interaction (see Appendix A).

The idea of a thereness system may be generalized to the setting of interacting processes.

**Definition 5**  (Generalized Thereness System)**.** *Let $\left\{\Pi_{i}\right\}$ be a collection of processes, $G(\Pi_{i})$ an interaction graph over these processes, and $O_{G}$ the set of all actual occasions created by $G(\Pi_{i})$. Assume the existence of a collection of auxiliary spatial distinctions and an equivalence relation $ADS(\left\{\Pi_{i}\right\})$ over the collection of actual occasithe properryons defined by $\Pi_{i}\equiv \Pi_{j}$ if they are indistinguishable under the auxiliary spatial distinctions. A generalized thereness relation on $G(\Pi_{i})$ is a collection of subsets $\left\{T_{x}^{k}|x\in O_{G}\right\}$ satisfying the thereness conditions and the following additional conditions:*

*1.*  *For every subprocess $\Pi_{i}$ with action set $A^{\Pi_{i}}$, and with $O_{\Pi_{i}}$ being the collection of all actual occasions generated by $\Pi_{i}$, the collection $\left\{T_{x}^{a_{j}}|a_{j}\in A^{\Pi_{i}},x\in O_{\Pi_{i}}\right\}$ forms a thereness system.*
*2.*  *For all i and for all actual occasions $x\in O_{\Pi_{i}}$ and actions $a_{j}\in A_{\Pi_{i}}$, there exists a k such that $T_{x}^{a_{j}}\subset T_{x}^{k}$.*
*3.*  *For all actual occasions $x,y\in O_{G}$, if $T_{x}^{k}=T_{y}^{k}$ for all k, then x≢y.*

The spacetime graph associated with a nexus of processes can be constructed by means of the generalized thereness system, but, in addition, we have spacetime graphs associated with each component through their individual actions. The above conditions ensure consistency so that the component spacetime graphs will be subgraphs of the nexus spacetime graph. Note that the spatial distinctions arrived at through the actions may need to be expanded through the spatial distinctions derived from the equivalence relation ADS(G).

Let the set of actions of process $\Pi_{i}$ be $\left\{a_{j}^{\Pi_{i}}\right\}$. For each process $\Pi_{0}$ in $G(\Pi_{i})$, consider the subgraph $G_{⊗}\left(\Pi_{0}\right)$ consisting of all product labeled edges $\Pi_{0}\overset{⊗}{-}\Pi_{k}$. We may construct the set of actions of the form $a_{0}^{\Pi_{0}}a_{1}^{\Pi_{k_{1}}}\cdots a_{n}^{\Pi_{k_{n}}}$ with $\Pi_{0}\overset{⊗}{-}\Pi_{k}\in G_{\prod }\left(\Pi_{0}\right)$. The order does not matter as these actions are assumed to be applied simultaneously (see the definition of ⊗ in Appendix A), but not all products may be permissible. If two or more actions may not be simultaneously carried out, then this product action must be excluded from the interaction action set. For any actual occasion $x^{\Pi_{k_{l}}}$ of process $\Pi_{k_{l}}$ in the prior generation, the action of an interaction product on this actual occasion is given by $x^{\Pi_{k_{l}}}a_{0}^{\Pi_{0}}a_{1}^{\Pi_{k_{1}}}\cdots a_{n}^{\Pi_{k_{n}}}=x^{\Pi_{k_{l}}}a_{l}^{\Pi_{k_{l}}}$.

This is not quite precise, however, because each component process $\Pi_{k}$ may be interacting with additional processes, so we actually need to consider the connected component of $G(\Pi_{i})$ containing $G_{⊗}\left(\Pi_{0}\right)$.

If an edge $\left\{\Pi_{0},\Pi_{j}\right\}$ is labeled by a ⊕ operator, then one may include any operator from $G_{\prod }\left(\Pi_{0}\right)$ and from $G_{\prod }\left(\Pi_{j}\right)$, but, as these have already been included, we see that the ‘plus’ edges can be ignored.

We now extend the action-based thereness system associated with single processes to an interaction nexus.

**Theorem** **5** (Nexus Action-Based Thereness System)**.** *Let $\left\{\Pi_{i}\right\}$ be a set of processes involved in an interaction nexus described by an interaction graph $G(\Pi_{i})$, and let $\hat{A}$ be a collection of product action operators $\hat{A}=\left\{a_{0},a_{1},a_{2}\ldots \right\}$, each of the form $b_{0}^{\Pi_{0}}b_{1}^{\Pi_{1}}\cdots b_{n}^{\Pi_{n}}$ for some processes in $\left\{\Pi_{i}\right\}$, with each $b_{j}$ an action from the action set of process $\Pi_{j}$, and $\left\{\Pi_{0},\Pi_{k}\right\}$ is an edge in $G_{\prod }\left(\Pi_{i}\right)$ for k=−1,…n. A thereness class $T_{x}^{a_{i}}=\left\{x,xa_{i},xa_{i}a_{i},xa_{i}a_{i}a_{i},\ldots \right\}=\left\{x,xa_{i},xa_{i}^{2},xa_{i}^{3},\ldots \right\}$. $\hat{A}$ is called a generator of a thereness system, and the set of all thereness classes forms a thereness system if the following conditions hold:*

*1.*  *$X_{x}=x\hat{A}$ for all actual occasions x.*
*2.*  *For all informons x and actions $a_{i}$ and generations n, if x lies in generation n, then $xa_{i}$ lies in generation n+1.*
*3.*  *There is a unique operator, $a_{0}$, such that $a_{0}a_{i}=a_{i}a_{0}$.*
*4.*  *For all actual occasions x,y and any $a_{i}$, $xa_{i}=ya_{i}$ implies that x=y.*
*5.*  *For all informons x and actions $a_{i},a_{j}$ and any k>0, $xa_{i}^{k}=xa_{j}^{k}$ implies that i=j.*
*6.*  *For all actual occasions x and actions $a_{i}$, there exists an action $a_{-i}$ (called the reverse of $a_{i}$) such that $xa_{i}a_{-i}=xa_{-i}a_{i}=a_{0}^{2}$.*

As before, a straightforward proof shows that this results in a thereness system. Moreover, it also meets the conditions above for a generalized thereness system.

As defined, this thereness system applies to actions on single action occasions. However, this ignores the correlations that are induced by the interaction nexus on the generation of actual occasions. Thus, we also need to consider the idea of a generalized actual occasion, being a set of actual occasions that are acted upon simultaneously by the interaction nexus. Such a generalized actual occasion takes the form $\left\{x^{\Pi_{1}},\ldots ,x^{\Pi_{n}}\right\}$ for some processes $\Pi_{i}$, where each $x^{\Pi_{i}}$ is an actual occasion of the single process $\Pi_{i}$. An action on such a generalized actual occasion takes the form $a_{1}^{\Pi_{1}}\cdots a_{n}^{\Pi_{n}}$, where $a_{i}^{\Pi_{i}}$ is an action of the single process $\Pi_{i}$. One can define an action-based thereness system on these generalized actual occasions and generalized actions in the obvious manner to form a generalized nexus action-based thereness system.

This seems unduly complicated, but, in a process nexus, interactions may result in correlations in the generation of actual occasions that would not be apparent from the consideration of single actual occasion-based spacetime histories alone. This is certainly true of entanglement situations, in which the single-process spacetime histories (and associated probabilities) make it appear as though these component processes are independent (the marginal probabilities for the component processes are identical to those when they are treated as independent), whereas the generalized thereness space shows that the generation of these actual occasions is in fact highly correlated. These generalized thereness spaces are thus important in understanding the appearance of contextuality in the probabilistic structure of these process dynamics.

If we now were to construct spacetime histories for all possible evolutions of an interaction nexus from its initiation to its termination, we would have a (very large) collection of (very large) finite graphs. In principle, at least, it should be possible to estimate or determine the frequency of appearance of various actual occasion configurations; thus, a probabilistic structure can emerge. In particular, one may determine the probabilities for the use of different actions upon a given collection of prior actual occasions and determine a transition preference graph for the nexus [73]. These transition probabilities may then be used to determine whether or not contextuality might appear in the nexus dynamics. The presence of obstructions to particular combinations of actions may result in losses of completeness or of transitivity in the associated transition preference graph—a pre-condition for the appearance of contextuality [73]. Thus, these spacetime histories may prove useful in the search for contextuality in both classical and quantum systems.

## 11. Discussion and Conclusions

Time and space are fundamental concepts, not just in science but in our everyday lives, yet they still seem mysterious and inscrutable. Regardless of whether one believes in a block universe or some version of presentism, events still tend to be viewed as having a place *in* time and space, whether they happened, happen, or will happen then and there or simply *are*. Events may be of finite duration yet nevertheless are viewed as if sempiternal or eternal, especially to those who hold to a block universe view.

The process viewpoint stands in stark contrast: events happen; they happen only at the time at which they happen and not at any other time. After they happen, they cease to exist. The most primitive events—Whitehead’s actual occasions—come into being, persist only long enough to pass information on to the next generation, and then fade away. In a process reality, events are *generated*, and so are time and space. As described previously, the process view of time [4,5] is one of local becoming, linked to each individual process, as proposed by Arthur [18], and fragmented, similar to that proposed by Fine [54] and Iaquinto and Torrengo [55].

The concept of process includes a vast array of entities, including those studied in the biological, social, and economic sciences and not just the physical sciences, so the concept of time from a process perspective is broadened to a notion of temporal distinction, being something that marks the presence of change. Likewise, the process concept of space is also much broader than that of physical space, constituting the notion of spatial distinction, being something that makes possible counting or the separability of entities. Separability is distinct from distinguishability (which is the ability to say that something is this thing) and is the ability to say that one has this thing *and* that thing, as opposed to just this thing, regardless of whether or not one can assert which is which. For example, fundamental particles may be indistinguishable but are usually separable. Spatial and temporal distinctions are informational and relational in nature.

In [4,5], it was argued that the temporal structures generated by processes take the form of an ordered chain of anti-chains, with each anti-chain representing a distinct generation of actual occasions, which can be represented as a directed graph with edges only appearing between adjacent anti-chains, which correspond to prior nascent generation pairs.

In this paper, it is shown that process actions (meaning the creation of and propagation of information to actual occasions) results in spacetime structures that can be described using labeled mixed graphs, which involve both directed and undirected edges. The directed subgraph of this mixed graph has the form of a graph of temporal distinctions, as described above. The undirected subgraph is a union of disconnected graphs, with each connected component lying within a separate anti-chain in the graph of temporal distinctions. These anti-chains are only connected by directed edges, while undirected edges only connect actual occasions within a single generation. The directed edges are labeled by the actions that they represent, while undirected edges lie between actual occasions that share a common prior actual occasion and are labeled by the pair of actions that propagate information from that prior to the two nascent actual occasions; in other words, $x\overset{\left(n,m\right)}{-}y$ if and only if there is an actual occasion *z* such that $z\overset{n}{\to }x$ and $z\overset{m}{\to }y$.

In the process algebra setting, these process spacetime structures are meant to be *interpreted* by means of mappings to more traditional mathematical structures—for example, causal manifolds. While an intrinsic notion of time is represented by the ordering of the anti-chains (generations), there is no requirement that, in an interpretation, all of the actual occasions that form a single generation must be assigned the same time coordinate in the interpretation structure. Only the order structure must be preserved. The actual occasions within an anti-chain possess no order relations and so they may be assigned a range of possible times. Thus, different observers may impose different interpretations with different spacetime coordinates being assigned. When the interpretation corresponds to a physical spacetime, it is obvious that directed edges correspond to timelike relations between the actual occasions that are their vertices. The undirected edges can be related to spacelike relations, but, more importantly, they may express correlations between the actual occasions that are their vertices—correlations that are lacking in the usual spacetime representations. Thus, the process spacetime potentially conveys more information about information flow, correlations, and causal relations than appears in our usual spacetimes.

The concept of location is an emergent feature of process spacetimes, not an intrinsic feature. The emergent concept of positions, formed of equivalence classes, each representing a particular location, corresponds to the usual notion of a spatial coordinate. However, an interpretation could well assign different spatial coordinates to the elements of a position class—in the process algebra, it is only the temporal ordering that must be preserved under the interpretation embedding. If positions are preserved, then it is clear that the embedding of the process spacetime Π(X) into an interpretation M will factor through the space T⊗P, where T is a time space and P is the space of positions. This shows that time and space are truly intertwined in a process spacetime.

A process spacetime is absolute relative to the process that generates it. Although no information ever passes between the actual occasions that form a single generation, if the directed edges are interpreted using some metric, then a distance can be inferred for each of the undirected edges. However, no such inference of ‘distance’ need, in general, be possible between arbitrary actual occasions. Moreover, if there are two process spacetimes for independent processes, then no inference is possible unless either a signal passes between them so as to establish a common standard or it is possible to trace back along the generations to some common prior process and then extrapolate forward. If no such common ancestor or signal exists, then these two process spacetimes are themselves independent of one another, again showing that this global spacetime may be fragmented. Of course, it may be possible for some external observer to impose a relationship between them by virtue of some interpretation, but this knowledge may be unavailable within the nexus of processes.

One point needs to be emphasized. The actions of a process will generate a single process spacetime, which represents the actuality generated by that process. It is not a container for events, nor is it a set of potentiae for all possible events. It is merely one instance of the generation of events by one process. Repeating the process action may result in a different process spacetime. Taking the graphical sum of all possible evolutions creates a space of potentiae for the given process. If the process has many variations due to changes in internal parameters, one may repeat this procedure for all possible values of these parameters and obtain a superspace of potentiae. An interpretation of this process-covering space would seem to be a plausible candidate for some form of container spacetime. However, it should be noted that this—possibly infinite—container spacetime or space of potentiae is not ‘real’ but rather is a heuristic construction, which is consistent with Whitehead’s view that infinite (continuous) structures represent sets of potentiae and not of actualities.

The labels on the edges of a process spacetime may have multiple interpretations. Thus, they might represent a generalized concept of velocity, or one of proper time, or a correlation. Mathematically, it is interesting to ask what interpretations are possible for a given process spacetime. This is a subject for future study. In particular, it is interesting to ask which process spacetimes admit interpretations via Minkowski or more general causal manifolds. Much work needs to be undertaken to address the question of how a process spacetime structure changes for different external observers and how it appears to the participants in a nexus of processes.

It has been shown that, if the actions of a process commute with one another, then the process spacetime will possess an intrinsic lattice structure. This raises another set of interesting mathematical questions regarding the relationship between the algebraic structure of the monoid of process actions and the geometric, order, and graphical structure of the generated process spacetimes. One often sees terms such as commutative or non-commutative geometry used in the context of Hilbert spaces and their operators, but, here, we see a more concrete relationship between an algebraic structure and a geometric structure.

Finally, it has been suggested that the undirected edges linking actual occasions within a generation, being related to those edges having a common prior actual occasion, may represent information about correlations between those associated actual occasions. This is an interpretation that is currently under active investigation and connects with a larger project directed at seeking to understand the constraints on the dynamics of systems that are necessary and sufficient for the appearance of true or Type II contextuality [24] among various states of the system [73]. The belief that Type II contextuality was unique to quantum mechanics meant that much research focused upon the structure of the space of measurement operators and the associated probabilities. The discovery that Type II contextuality can occur in classical settings has expanded this research to consider context-dependent Kolmogorov probabilities as well [24]. However, missing from this is an understanding of the relationship between the underlying dynamics of systems and the presence of contextuality. Research has revealed that classical deterministic systems cannot exhibit Type II contextuality [75]; thus, only non-deterministic or stochastic systems can do so. It should be noted that the demonstration of Type II contextuality in both physical and psychological experiments demonstrates that these systems *must* be either non-deterministic or stochastic in nature [75]. Complicating the matter is the fact that many systems of interest in the biological and social sciences do not possess probabilities in a strict sense, perhaps due to non-stationarity, to the presence of singular events, or to being non-deterministic, so that any apparent probabilities are not intrinsic to the dynamics but are instead emergent from the dynamics. This has led to research examining the relationship between system dynamics and emergent probabilities employing the concept of a dynamical preference graph [73]. Closely related is research on graphical approaches to contextuality [76,77], but the dynamical preference approach takes a different perspective. Previous work has highlighted the importance of incompleteness and intransitivity in these dynamical preference graphs as necessary, albeit not sufficient, conditions for Type II contextuality. The current work on process spacetime provides another approach to exploring these dynamical preference graphs and thus contextuality in a broader range of systems. This is important for the study of contextuality in quantum mechanics, but it is more important for understanding the appearance of contextuality in classical systems, such as in psychology [25] and economics [26,27,29].

The process algebra approach thus would appear to be a fruitful source of interesting mathematical and physical applications—within quantum mechanics but also within biology, psychology, economics, and the social sciences.

## Data Availability

No new data were created or analyzed in this study. Data sharing is not applicable to this article.

## Appendix A. Process in the Process Algebra

The definitions included in this appendix are taken verbatim from [3], where the process algebra is described in detail. A formal process generates actual occasions akin to the manner in which combinatorial games [78,79] are used to create mathematical structures in mathematical logic (model theory) [80,81,82]. An actual occasion is created in a series of short rounds in which information is propagated, while a generation is created in a series of rounds, thus creating a set of actual occasions. Processes can be either *active*, thus generating actual occasions, or *inactive*, meaning that they are merely potential in nature. If P denotes an active process, then $\bar{P}$ denotes its inactive form.

Formal processes generate abstract *informons*, distinguishing them from actual occasions, which occur in reality; for simplicity, I use the term actual occasion here as well.

An abstract actual occasion possesses intrinsic components, which represent concrete aspects, and extrinsic components, which represent abstract aspects or perhaps contributions from some observer. An actual occasion has the form

$$\left[n\right]<m_{n},\phi_{n};p_{n},\Gamma_{n}>\left\{G_{n}\right\}.$$

1. *n* is a heuristic label.
2. $p_{n},\Gamma_{n}$ are *intrinsic* components. $p_{n}$ is a vector of properties that links the actual occasion to its generating process. It too is heuristic. $\Gamma_{n}$ is the information associated with the actual occasion, which is propagated and incorporated into nascent actual occasions by the generating process. $\Gamma_{n}$ is a feature of the actual occasion itself and is represented by an element in some mathematical structure (consider the representation of various fundamental particles, e.g., scalar (Higgs), vector (photon), spinor (electron), or tensor (graviton), for example).
3. $m_{n},\phi_{n}$ are the *extrinsic* components imposed by an external observer or process (real or imagined for heuristic purposes). $m_{n}$ is a map from each actual occasion to some formal causal space M (for example, a manifold with a pseudo-metric ρ and compatible partial order). $\phi_{n}$ is a mapping on M belonging to a Banach, Hilbert, or other function space, which is denoted H(M). It provides an interpolation [46] from or interpretation of the information of the actual occasions.
4. $G_{n}$ is a causally ordered set of prior actual occasions called the *content* of the actual occasion *n*. It consists of all prior actual occasions that propagated information that was (or will be) incorporated into *n*. It is also introduced for heuristic purposes, but it does convey important information regarding the process.

A formal process is modeled (purely heuristically) as a two-person combinatorial game [78,79], as utilized in the construction of models and relation algebras in mathematical logic [80,81,82]. Moves consist of actions, which, starting from a prior actual occasion, either initiate the creation of a nascent actual occasion or propagate information from the prior to a nascent actual occasion. These games are characterized by several parameters:

1. *N*, the total number of actual occasions in each generation.
2. $\bar{R}$, the total number of rounds per generation cycle.
3. *R*, the number of actual occasions generated in each round. Hence, $N=R\bar{R}$.
4. *s*, the number of short rounds per round.
5. *r*, the number of prior actual occasions, which may condition the creation of a single actual occasion. Often, we will assume that s=Rr, so *s* can be dropped.
6. $r^{\prime }$, the number of nascent actual occasions to which each prior actual occasion may contribute as a condition (in many cases, $r=r^{\prime }$ or $r=r^{\prime }=N$).
7. p, the set of properties disposed by the process—often, p will consist of a set of subsets, with each subset corresponding to a specific property and the elements of each subset corresponding to the measured values so disposed.
8. S, the strategy utilized by the process in its acts of prehension and concrescence.
9. $t_{\Pi }$, which sets the temporal scale between generation cycles.
10. $l_{\Pi }$, which sets the spatial scale between actual occasions within a causal tapestry.
11. A process may be denoted as ${}_{r^{\prime }}^{N}\Pi_{r}^{R}\left(p,S,t_{\Pi },l_{\Pi }\right)$.

In general, the parameters $N,R,\bar{R},r,r^{\prime },s,S,t_{\Pi },l_{\Pi }$ will be fixed for a given process and only the properties will vary. In this case, we will refer to a process as $P_{p}$ for simplicity.

A process is said to be *primitive* if R=1; otherwise, it is *complex*.

Information propagation is strictly causal and local, thus being consistent with special relativity.

The simplest process is the zero process, $O=\bar{O}$, which, as one might imagine, does nothing at all.

*Histories* of a process are represented by sets of actual occasions called *causal tapestries*. A causal tapestry is a set of actual occasions, which can represent a round, a generation, or a history of generations.

**Definition** **A1.**
*A causal tapestry C is a tuple (I,F,G,P,p,d,M,I,Φ,ρ).*

1. *I is a set of actual occasions.*
2. *F is a directed graph with vertices I, called the information flow.*
3. *G is a non-directed graph with vertices I, called the skeleton.*
4. *P is a set of processes.*
5. *p is a map from F∪G→P such that, if (x,y)∈F and either {x,z} or {y,z} lies in G, then p((x,y))=p({x,z})=p({z,y}).*
6. *d is a signed metric on I such that, if (x,y)∈F, then d(x,y)≥0, and, if {x,y}∈G, then d(x,y)<0. This is optional, depending upon the processes.*
7. *M is a causal structure with causal order ⪯, pseudo-metric ρ, and value space I.*
8. *m:I→M, which is called the M interpretation.*
9. *The mapping $\Phi \left(m\right)=\sum_{n\in I}\phi_{n}\left(m\right)$ is called the global H(M) interpretation. It is thus an interpolation lying within a function space whose base space is M.*
10. *(n,m)∈F implies $m_{m}⪯m_{n}$.*
11. *For all n,m∈I, $d\left(n,m\right)=\rho \left(m_{n},m_{m}\right)$.*

*F* represents the flow of information induced by the processes that generated the causal tapestry, while *G* associates the actual occasions that comprise a single generation. *G* conveys topological or metric—but not causal—relations between the actual occasions of a generation.

The description of interactions was inspired by the different ways in which combinatorial games can be played [78,79]. The process algebra accounts for three fundamental aspects of generation.

1. The relative timing of the generation of actual occasions, which may be *sequential*, i.e., alternating, possibly randomly, from one process to another, or *concurrent*, i.e., actual occasions corresponding to each process are generated together.
2. The flow of information from prior to nascent actual occasions, which may be *exclusive*, where information propagates only among actual occasions corresponding to the *same* process, or *free*, where information may propagate among actual occasions corresponding to different processes.
3. The relationship between processes, which may be *independent*, *coupled*, *entangled*, or *interacting*.

The process algebra has 11 operators, reflecting the nuances of these different modes of interaction. They are as follows:

1. Independent: ,
2. Coupled: *
3. Succession (concatenation): ·
4. Free sequential (free sum): $\hat{⊕}$
5. Exclusive sequential (exclusive sum): ⊕
6. Free concurrent (free product): $\hat{⊗}$
7. Exclusive concurrent (exclusive product): ⊗
8. Free sequential interactive (free interactive sum): $\hat{⊞}$
9. Exclusive sequential interactive (exclusive interactive sum): ⊞
10. Free concurrent interactive (free interactive product): $\hat{⊠}$
11. Exclusive concurrent interactive (exclusive interactive product): ⊠

The basic rules for applying these operations in combining processes are the following:

1. The independent operator is used when two or more processes act completely independently of one another.
2. * is used when two independent processes begin generating actual occasions within the same spatial region and must ensure that they do not attempt to generate actual occasions at the same location. The processes ‘sense’ each other’s actual occasions without exchanging information.
3. Concatenation is used to designate the progression of processes generation by generation (thus marking changes in state and progression in time).
4. The free sum is only used for single systems and combining states that possess identical property sets (pure states).
5. The exclusive sum is used for single systems and combining states that possess distinct property sets (mixed states).
6. The free product is used for multiple systems that possess distinct characteristics (scalar, spinorial, vectorial, and so on), such as coupling a boson and a fermion. It is unclear whether two bosons might couple via a free product.
7. The exclusive product is used for multiple systems that possess the same characteristics, such as coupling two bosons or two fermions.

The three connectors—‘,’, ‘*’, and ‘·’—do not describe any information flow between processes (although · reflects a causal relationship between antecedent and subsequent processes) and serve more as logical connectives.
